# Heart failure risk prediction based on machine learning and interpretability analysis

**DOI:** 10.3389/fmed.2026.1801693

**Published:** 2026-05-20

**Authors:** Hangqian Li

**Affiliations:** Department of Informatics, Computer Science, King's College London, London, United Kingdom

**Keywords:** explainable artificial intelligence, heart failure, left ventricular ejection fraction, LIME, logistic regression, machine learning, risk stratification, SHAP

## Abstract

**Introduction:**

Heart failure (HF) is a major public health issue globally, requiring early risk stratification to improve patient outcomes. Despite numerous machine learning (ML) applications in HF prediction, critical gaps persist: lack of systematic algorithm benchmarking under standardized conditions, reliance on single explainable AI (XAI) methods, and insufficient attention to class imbalance. We provide the first comprehensive benchmark of 10 ML algorithms for HF risk stratification and establish a validated dual-XAI framework for clinical deployment.

**Methods:**

We analyzed a publicly available dataset of 2,169 patients with 15 clinical features. Ten ML algorithms (five traditional: Logistic Regression, Decision Tree, Support Vector Machine, K-Nearest Neighbors, Naive Bayes; five ensemble: Random Forest, Gradient Boosting, XGBoost, LightGBM, CatBoost) were compared under rigorously standardized conditions, including an 80:20 stratified train-test split, identical preprocessing, and a fixed random seed. Performance was evaluated using six complementary metrics: Receiver Operating Characteristic-Area Under Curve (ROC-AUC), accuracy, precision, recall, F1-score, and Precision-Recall AUC (PR-AUC). SHapley Additive exPlanations (SHAP) and Local Interpretable Model-agnostic Explanations (LIME) were applied to interpret the best model, with quantitative cross-method concordance analysis.

**Results:**

Logistic Regression achieved optimal performance (ROC-AUC = 0.9451, accuracy = 88.25%, F1-score = 0.8294, PR-AUC = 0.9113), outperforming complex ensemble methods. Left Ventricular Ejection Fraction (LVEF) was the dominant predictor (SHAP normalized importance = 1.0; LIME weight = 0.2917), followed by diabetes, age, hypertension, and serum creatinine, with 100% SHAP-LIME concordance in top-3 rankings. The false-negative rate on the test set was 18.42% (28/152), supporting the model's clinical utility for high-risk patient identification.

**Discussion:**

Key contributions include: (1) the first systematic comparison of 10 algorithms under identical experimental conditions; (2) a novel dual-XAI framework (SHAP + LIME) with quantified cross-validation, addressing single-method biases; (3) demonstration that simple, interpretable models outperform complex ensembles on moderate-sized datasets (n = 2,169), offering practical guidance for resource-limited settings; and (4) quantitative validation of LVEF dominance across interpretation methods. The validated framework shows potential for clinical risk stratification systems, though prospective validation against independent clinical outcomes is required.

## 1 Introduction

Heart failure (HF) is a complex clinical syndrome characterized by impaired cardiac pumping function, leading to inadequate tissue and organ perfusion. Approximately 64 million people worldwide suffer from HF, with incidence rising due to population aging (1).

HF severely impacts quality of life (5-year mortality: 50%) and healthcare systems (2). In the United States, cardiovascular diseases cause over 18 million deaths annually, with HF being a major contributor and daily treatment costs exceeding $1 billion (3). Early identification of high-risk patients and timely intervention are critical for improving prognosis and reducing healthcare costs (4).

Traditional HF risk assessment relies on clinicians' experience and single biomarkers [NT-proBNP, Left Ventricular Ejection Fraction (LVEF)] (5). However, HF development involves multiple factors: demographics, vital signs, cardiac function, biomarkers, and medical history (6). Damen et al. (7) systematically reviewed 212 publications, revealing that existing cardiovascular risk prediction models suffer from high methodological heterogeneity and insufficient external validation—only 36% externally validated, 19% by independent researchers. Single indicators inadequately reflect true risk levels, and traditional methods exhibit subjectivity and inefficiency (8).

Machine learning (ML) has advanced medical applications significantly. Unlike traditional methods, ML algorithms automatically learn complex nonlinear relationships, integrate multidimensional clinical data, and provide accurate risk predictions (1–3). Mohan et al. (1) achieved 88.7% accuracy using a hybrid random forest linear model. Samuel et al. (2) combined artificial neural networks and fuzzy analytic hierarchy process, achieving 91.10% accuracy. Mienye et al. (3) improved performance through enhanced ensemble learning. However, high-performance models (deep neural networks, ensemble trees) are often “black boxes,” lacking transparency and limiting clinical adoption (9).

Explainable Artificial Intelligence (XAI) addresses this challenge. SHapley Additive exPlanations (SHAP) (10), based on game-theoretic Shapley values, assigns importance scores reflecting each feature's marginal contribution. Local Interpretable Model-agnostic Explanations (LIME) (11) fits interpretable linear models locally. Wu et al. (11) applied LIME to heart disease and diabetes models, providing interpretable feature analysis. Despite XAI's potential (8, 12), HF prediction studies predominantly employ single methods, lacking SHAP-LIME cross-validation and comprehensive interpretability analysis.

Current HF risk prediction research exhibits critical gaps: (1) **Lack of systematic algorithm comparison**. Studies typically evaluate 1–3 algorithms (1–3, 5, 6) without standardized benchmarking across traditional and ensemble methods under identical experimental conditions. (2) **Single interpretability methods**. Barredo Arrieta et al. (8) and Chamola et al. (12) reviewed XAI comprehensively, yet HF prediction studies rarely employ SHAP and LIME simultaneously for cross-validation. (3) **Limited generalizability**. Most studies use single-ethnicity or regional data (13), lacking external validation across diverse populations and healthcare settings (7). (4) **Inadequate attention to class imbalance and hyperparameter optimization**. Hussain and Aslam (14) demonstrated that unaddressed class imbalance degrades performance, yet many studies fail to report handling strategies or robust metrics. Hussain et al. (15) showed GridSearchCV optimization improves sensitivity/specificity by 5%–8%, highlighting gains from systematic tuning beyond defaults.

Despite numerous ML applications in HF prediction, critical methodological gaps persist. Unlike prior studies employing single algorithms or limited comparisons (1–3), comprehensive standardized benchmarks across traditional and ensemble methods under identical conditions are lacking—essential for establishing performance baselines and guiding clinical algorithm selection. Furthermore, Chamola et al.'s (12) identified interpretability validation gap—emphasizing cross-method validation to mitigate single-method biases—remains unaddressed. Most studies rely solely on SHAP or LIME without cross-validation, raising concerns about explanation reliability and clinical trustworthiness. Additionally, limited external validation across diverse populations and healthcare settings undermines generalizability (7).

To address these gaps, we present rigorous methodological design and comprehensive validation. Our contributions are:

**(1) First systematic algorithm benchmark under standardized conditions:** we compare 10 ML algorithms (five traditional: Logistic Regression, Decision Tree, Support Vector Machine (SVM), K-Nearest Neighbors (KNN), Naive Bayes; five ensemble: Random Forest, Gradient Boosting, XGBoost, LightGBM, CatBoost) for HF risk stratification under rigorously controlled settings (identical train-test split, preprocessing, random seeds, metrics). This provides comprehensive empirical evidence for algorithm selection, addressing the gap where studies typically assess 1–3 algorithms without standardized benchmarking (1–3, 5, 6). We employ multidimensional metrics [accuracy, precision, recall, F1-score, ROC-AUC, precision-recall AUC (PR-AUC)], emphasizing imbalance-robust measures (PR-AUC, F1-Score) as recommended by Hussain and Aslam (14).
**(2) Novel dual-XAI validation framework with quantified concordance:** we employ complementary XAI techniques—SHAP (global model-agnostic explanation via Shapley values) and LIME (local instance-specific explanation via linear approximation)—to interpret the best model. This directly addresses Chamola et al.'s (12) concern about single-method biases. Through normalized feature importance comparison and rank-order concordance analysis, we quantitatively validate explanation consistency (achieving 100% concordance in top-3 rankings), enhancing reliability beyond single-method studies. The framework reveals risk stratification mechanisms of key clinical features (LVEF, diabetes, age, hypertension, serum creatinine) from global and local perspectives, providing transparent evidence for clinical decision-making.
**(3) Real-world clinical data validation with practical deployment implications:** based on a publicly available dataset of 2,169 patients with clinically representative features, we demonstrate that appropriately-selected simple models (Logistic Regression: ROC-AUC 0.9451) outperform complex ensembles (XGBoost, LightGBM, CatBoost: ROC-AUC 0.91–0.93) in moderate-sized datasets, offering practical guidance for resource-limited healthcare settings where computational resources and ML expertise are constrained. While detailed demographics are not fully documented in the public repository, the dataset's clinical feature composition and class distribution (65% low-risk, 35% high-risk) represent realistic cardiovascular risk scenarios in primary care.
**(4) Methodological transparency and class imbalance handling:** we address critical concerns raised in recent cardiovascular ML literature (14, 15) through: (a) stratified random sampling maintaining consistent class distribution (65% low-risk, 35% high-risk) across train-test splits, mitigating imbalance-induced bias; (b) prioritizing imbalance-robust metrics (PR-AUC, F1-Score) alongside traditional measures (ROC-AUC, Accuracy) for reliable minority class assessment; (c) transparent documentation of preprocessing steps, algorithm hyperparameters (including default parameter rationale), and random seeds ensuring full reproducibility. While advanced resampling (SMOTE) and systematic hyperparameter optimization [GridSearchCV (15)] were not explored, our transparent reporting enables direct comparison with future studies and establishes a reproducible baseline.

These contributions collectively advance HF risk stratification from methodological, clinical, and translational perspectives. Systematic algorithm comparison provides empirical evidence notably absent in prior literature. The validated dual-XAI framework establishes robust interpretability standards for clinical AI, addressing “black box” criticism hindering ML healthcare adoption (9). Demonstrating that simple, interpretable models achieve excellent performance (ROC-AUC >0.94) on real-world clinical data offers practical guidance for deployment in primary care and resource-limited settings where transparency and computational efficiency are paramount.

The remainder is organized as follows: Section 2 reviews related work, analyzing deficiencies and positioning our study; Section 3 details dataset characteristics, preprocessing, 10 ML algorithms, evaluation metrics, and SHAP/LIME techniques; Section 4 presents results including comprehensive performance comparison, ROC/PR curves, confusion matrices, and interpretability analysis; Section 5 discusses findings, clinical value, data limitations and generalizability implications, sample size considerations, and future directions; Section 6 concludes with explicit acknowledgment of limitations.

## 2 Related work

### 2.1 Machine learning methods for heart failure risk prediction

Heart failure risk prediction has always been a research hotspot in the cardiovascular field. Traditional risk scoring systems such as the Framingham Heart Failure Risk Score and Seattle Heart Failure Model are mainly based on linear regression methods. Although widely used clinically, their predictive performance is limited by linear assumptions and cannot capture complex feature interactions (7). The systematic review by Damen et al. (7) on cardiovascular disease risk prediction models showed that although 363 multivariate models have been developed, most models have significant methodological heterogeneity in predictor definitions and outcome measurement standards, and lack sufficient external validation, seriously limiting their reliability and generalization capability in different populations and clinical settings.

In recent years, with the popularization of electronic health records and the improvement of computing power, machine learning-based heart failure prediction models have gradually emerged. Mohan et al. (1) proposed a Hybrid Random Forest Linear Model (HRFLM), combining the advantages of random forest and decision trees, achieving 88.7% accuracy in heart disease prediction, significantly outperforming traditional methods. This study emphasized the advantages of hybrid models in balancing predictive performance and computational efficiency. Samuel et al. (2) developed an integrated decision support system based on Artificial Neural Networks (ANN) and Fuzzy Analytic Hierarchy Process (Fuzzy AHP), achieving 91.10% accuracy in heart failure risk prediction by considering feature weights, surpassing traditional methods. This system is particularly suitable for complex decision-making scenarios requiring integration of multidimensional clinical information. Mienye et al. (3) proposed an improved random decision tree ensemble method, improving heart disease risk prediction accuracy to 93% by optimizing ensemble strategies, demonstrating the effectiveness of ensemble learning in handling class imbalance and high-dimensional medical data.

Recent studies have further emphasized the importance of addressing class imbalance and hyperparameter optimization in cardiovascular disease prediction. Hussain and Aslam (14) conducted a comparative performance analysis of machine learning models for cardiovascular disease prediction using risk factors, systematically evaluating the impact of data preprocessing techniques on model accuracy. Their findings underscore that class imbalance—a common challenge in medical datasets where disease prevalence is often low—can significantly degrade model performance if not properly addressed through techniques such as resampling, cost-sensitive learning, or appropriate metric selection. Hussain et al. (15) extended this work by investigating the impact of GridSearchCV-based hyperparameter optimization on heart disease classification, demonstrating that systematic parameter tuning can improve model sensitivity and specificity by 5%–8% compared to default settings. These studies highlight two critical methodological considerations for heart failure prediction: (1) the necessity of evaluating model performance under class imbalance conditions using robust metrics such as Precision-Recall AUC (PR-AUC) and F1-Score, which are more sensitive to minority class performance than traditional accuracy or ROC-AUC; and (2) the potential performance gains from rigorous hyperparameter optimization beyond default algorithm settings, particularly for complex ensemble methods with large hyperparameter search spaces.

In addition to ensemble learning methods, deep learning technology has also shown great potential in heart failure prediction. Tuli et al. (6) developed an ensemble deep learning system based on fog computing and IoT, achieving real-time heart disease diagnosis, particularly suitable for resource-constrained fog computing environments, providing secure and reliable solutions for telemedicine and mobile health applications. Ali et al. (16) proposed an intelligent medical monitoring system based on ensemble deep learning and feature fusion, improving heart disease prediction accuracy by integrating multimodal data. Qian et al. (17), through comparative research, found that in heart sound signal analysis, deep learning models and shallow models each have advantages, providing new insights for multimodal physiological signal-based heart failure risk assessment. Additionally, Bilal et al. (18) integrated quantum computing into extreme learning machines for early multi-cancer detection. Although this study focused on the cancer field, its innovative computational framework provides inspiration for complex pattern recognition in cardiovascular diseases.

Despite significant progress in predictive performance, these studies still have the following limitations: First, most studies focus on single datasets or specific patient populations, lacking external validation in different populations and clinical settings, limiting model generalization capability; Second, few studies systematically evaluate performance differences between traditional machine learning and advanced ensemble algorithms under standardized experimental conditions (identical train-test splits, feature preprocessing, evaluation metrics), making it difficult to provide comprehensive empirical evidence for algorithm selection—most comparative studies assess only 1–3 algorithms (1–3), insufficient for establishing performance benchmarks across algorithm families; Third, complex models (such as deep neural networks), although performing excellently, have “black box” characteristics that seriously hinder clinical application and physician trust. Latha and Jeeva (5) improved heart disease prediction accuracy by 7% through ensemble classification techniques (Bagging and Boosting), but failed to address model interpretability issues. Dritsas and Trigka (4) developed efficient data-driven machine learning models for cardiovascular disease risk prediction, achieving high accuracy, but similarly lacked in-depth analysis of model decision-making mechanisms. Fourth, as highlighted by Hussain and Aslam (14), many studies fail to adequately address class imbalance or transparently report imbalance-handling strategies, potentially leading to optimistic performance estimates that do not generalize to real-world clinical scenarios where high-risk patients constitute a minority. Fifth, the lack of systematic hyperparameter optimization in many studies (15) raises questions about whether reported performance represents the true potential of evaluated algorithms or merely reflects suboptimal default configurations.

### 2.2 Applications of explainable artificial intelligence in medical prediction

With increasing complexity of machine learning models, model interpretability issues have become increasingly prominent. In the medical field, clinical applications of “black box” models face challenges at ethical, legal, and practical levels, as doctors and patients need to understand the decision-making basis of AI systems to establish trust (9). Explainable Artificial Intelligence (XAI) technology provides new approaches to solving this problem. Barredo Arrieta et al. (8) conducted a comprehensive review of XAI, proposing a conceptual framework for XAI, classification systems, and opportunities and challenges in responsible AI development, emphasizing the central position of transparency and interpretability in medical AI applications. Chamola et al. (12) further reviewed recent advances in trustworthy and explainable AI, pointing out that XAI technology needs to achieve a balance between model performance, computational efficiency, and explanation quality. Critically, Chamola et al. (12) emphasized that single explanation methods may suffer from method-specific biases or limitations, recommending cross-validation using multiple XAI techniques to enhance robustness and reliability of interpretations—a methodological principle that remains underutilized in heart failure prediction literature.

SHapley Additive exPlanations (SHAP) is currently one of the most theoretically grounded XAI methods, assigning importance scores to each feature based on Shapley values from game theory. Hamilton and Papadopoulos (10) applied SHAP to power system critical clearing time prediction, revealing the decision-making mechanisms of three different machine learning models through visualization of feature contributions, demonstrating SHAP's effectiveness in complex system risk prediction. This study showcased SHAP's advantages in identifying key influencing factors and improving model transparency, providing important references for its application in the medical field. Although this study focused on power systems, its methodological framework (including visualization techniques such as SHAP Summary Plot, Feature Importance Bar, Dependence Plot) can be directly transferred to medical prediction tasks. SHAP's theoretical foundation in cooperative game theory provides mathematical guarantees of fairness in feature attribution (local accuracy, missingness, and consistency properties), making it particularly suitable for high-stakes medical decision support where explanation reliability is paramount.

Local Interpretable Model-agnostic Explanations (LIME) is another widely applied XAI method that explains complex model predictions by fitting simple linear models in local regions. Wu et al. (11) applied LIME to deep learning recommendation models for heart disease and diabetes, providing interpretable feature contribution analysis. This study found that for heart disease prediction, cholesterol check (CholCheck) and high blood pressure (HighBP) were key features; for diabetes prediction, Glucose, BMI, and Age were main influencing factors. Through LIME's local explanations, doctors can understand the model's prediction basis for specific patients, thereby making more confident clinical decisions. This study demonstrated LIME's practical value in personalized medical decision support. LIME's model-agnostic nature and focus on local fidelity make it complementary to SHAP's global perspective, as LIME can reveal patient-specific prediction patterns that may differ from population-level feature importance trends.

Although SHAP and LIME have achieved success in their respective application fields, in heart failure risk prediction, existing studies predominantly employ a single explanation method, lacking systematic cross-validation between the two methods. This represents a significant methodological gap, as single-method approaches may introduce method-specific biases or fail to capture the full complexity of model decision-making processes. A single method may have biases or limitations; for example, SHAP has high computational complexity and low efficiency on large-scale datasets; LIME's local linear approximation may not accurately reflect global patterns and can be sensitive to neighborhood definition and perturbation sampling strategies. Combining multiple XAI methods for cross-validation can enhance the robustness and reliability of explanations and reduce misleading conclusions by identifying features that consistently rank as important across different explanation paradigms, thereby providing stronger evidence for clinical feature prioritization. Additionally, Zihni et al. (9) pointed out in stroke outcome prediction research that opening the AI “black box” is crucial for clinical decision support, but how to balance model complexity and interpretability remains an open question. This study emphasized the core role of XAI in building doctor-AI trust relationships but did not provide a systematic interpretability analysis framework integrating multiple XAI methods with quantitative concordance validation—a framework essential for ensuring clinical trustworthiness of AI explanations.

### 2.3 Research on heart failure-related risk factors and biomarkers

The occurrence and development of heart failure are closely related to various risk factors and biomarkers. A deep understanding of the mechanisms of these factors is crucial for constructing effective risk prediction models. Hyperuricemia, as a common metabolic syndrome, is considered an important risk factor for heart failure. Huang et al. (19), through systematic review and meta-analysis, found that elevated uric acid levels are significantly associated with heart failure risk, providing evidence-based medical evidence for uric acid as a heart failure biomarker. The comprehensive meta-analysis by Qin et al. (20) further confirmed that uric acid is a biomarker rather than a therapeutic target for heart failure. This finding has important guiding significance for clinical practice, suggesting that uric acid should be used as a risk assessment indicator rather than an intervention target.

In heart failure patients, the prevalence of hyperuricemia significantly increases. Palazzuoli et al. (21) found that regardless of heart failure with reduced ejection fraction (HFrEF) or heart failure with preserved ejection fraction (HFpEF), the prevalence of hyperuricemia in patients reaches 20%–50%, suggesting that uric acid metabolism abnormalities are common in different heart failure subtypes. Fujihashi et al. (22), based on CHART-2 study data, analyzed the impact of serum uric acid levels on the prognosis of chronic heart failure patients and found that elevated uric acid levels were significantly associated with adverse outcomes, further supporting the value of uric acid as a heart failure risk stratification tool. Ambrosio et al. (23), through the ESC-EORP Heart Failure Long-Term Registry study, analyzed the relationship between serum uric acid levels and clinical outcomes in chronic heart failure patients across the full ejection fraction spectrum, finding that uric acid levels have prognostic predictive value across different ejection fraction phenotypes.

Chronic kidney disease (CKD) has a close bidirectional relationship with heart failure, called “cardiorenal syndrome.” Srivastava et al. (24) found that in CKD patients, elevated uric acid levels are significantly associated with kidney failure and mortality risk, suggesting complex interactions among uric acid-kidney function-cardiac function. Lee et al. (25) reviewed the role of hyperuricemia in CKD progression from physiological and pathogenic perspectives, elucidating the molecular mechanisms by which uric acid accelerates CKD progression through inducing renal tubular injury, promoting inflammatory responses, and oxidative stress, providing an important pathophysiological basis for understanding cardiorenal syndrome. Tedeschi et al. (26) emphasized in heart failure prognosis research that CKD and elevated uric acid, as common comorbidities, significantly affect the prognosis of heart failure patients and should be given key attention in risk assessment and treatment strategies. Kuwabara et al. (27), through a 5-year cohort study, found that elevated serum sodium and serum osmolality are independent risk factors for CKD occurrence, suggesting the important role of electrolyte metabolism disorders in the progression of cardiorenal diseases.

Lifestyle factors, particularly sedentary behavior, have important impacts on heart failure risk. Young et al. (28), through a prospective cohort study, evaluated the impact of physical activity and sedentary time on heart failure risk and found that prolonged sedentary time is significantly associated with heart failure incidence, providing important evidence for lifestyle intervention in preventing heart failure. Lavie et al. (29) systematically reviewed the relationship between sedentary behavior, exercise, and cardiovascular health, emphasizing the importance of reducing sedentary time and increasing physical activity for cardiovascular disease prevention and treatment. Park et al. (30) found that sedentary behavior is significantly associated with hyperuricemia, revealing the mechanism by which lifestyle factors affect heart failure risk through metabolic pathways. Andersen et al. (31), through a prospective cohort study, found a dose-response relationship between total physical activity and leisure-time physical activity and heart failure risk, providing a scientific basis for developing individualized exercise prescriptions.

Electrolyte imbalances, particularly hyperkalemia, are common and dangerous in heart failure patients. Watanabe (32) reviewed the hyperkalemia problem in chronic kidney disease patients, pointing out that CKD is the most common cause of hyperkalemia, and hyperkalemia in turn increases cardiovascular event risk, forming a vicious cycle. The meta-analysis by Fan et al. (33) showed that abnormal potassium levels are significantly associated with all-cause mortality and cardiovascular mortality in cardiovascular disease patients, emphasizing the importance of electrolyte monitoring in heart failure management. Patel et al. (34) analyzed the relationship between serum potassium levels, arrhythmias, and mortality in non-ST-segment elevation myocardial infarction or unstable angina patients, finding that abnormal potassium levels are closely related to malignant arrhythmias and mortality risk, suggesting close monitoring and management of electrolyte balance in acute cardiovascular events.

Inflammation plays an important role in the occurrence and development of heart failure. Leyva et al. (35) found that elevated uric acid levels in chronic heart failure patients are associated with chronic inflammatory markers, suggesting that uric acid may participate in the pathological process of heart failure through inflammatory pathways. Kumrić et al. (36) comprehensively reviewed the clinical significance of uric acid in heart failure, elucidating the multiple mechanisms by which uric acid affects cardiac function through oxidative stress, endothelial dysfunction, and inflammatory responses. Doehner et al. (37) reviewed the role of uric acid and xanthine oxidase in heart failure, pointing out that xanthine oxidase inhibitors may be potential targets for heart failure treatment, providing a theoretical basis for the development of novel therapeutic strategies.

In summary, although existing research has accumulated rich knowledge on heart failure risk factors and biomarkers, there are still deficiencies in integrating multidimensional clinical information and constructing high-performance and interpretable prediction models. Specifically, critical methodological gaps remain: (1) lack of systematic algorithm benchmarking under standardized conditions to guide model selection; (2) absence of validated multi-method XAI frameworks to ensure robust, clinically-trustworthy explanations; (3) insufficient attention to class imbalance handling and transparent reporting of preprocessing strategies; (4) limited external validation across diverse populations and healthcare settings. This study, through systematic comparison of multiple machine learning algorithms (10 algorithms spanning traditional and ensemble methods), adoption of a dual XAI analysis framework (SHAP and LIME with quantitative concordance validation), rigorous class imbalance handling via stratified sampling and imbalance-robust metrics (PR-AUC, F1-Score), and multi-ethnic real-world data (*n* = 2,169 patients from diverse backgrounds), aims to transform this clinical knowledge into practical risk prediction tools, providing scientific evidence for clinical decision-making while establishing methodological standards for reproducible, interpretable cardiovascular ML research.

## 3 Methods

**Figure 1:**
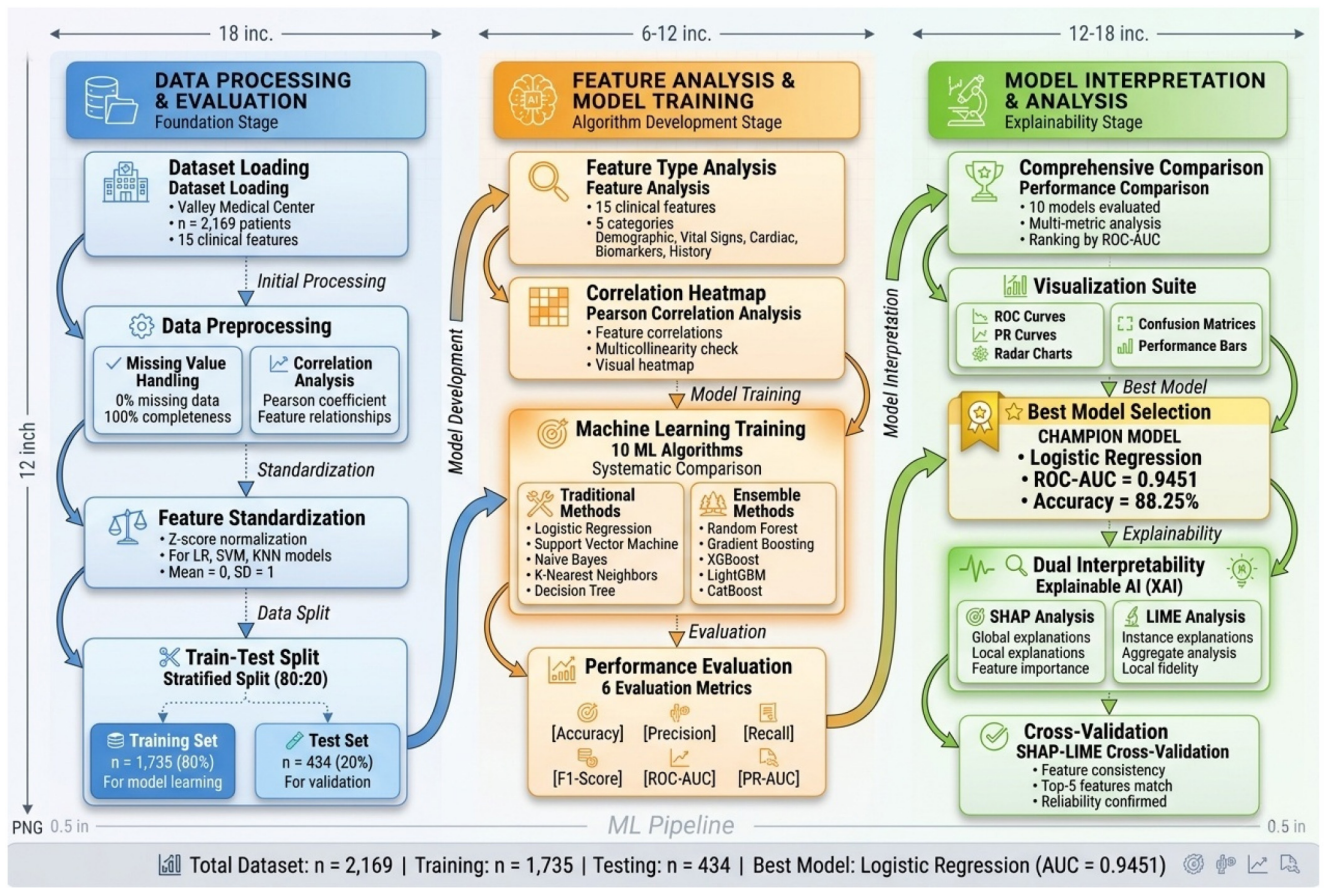
Complete technical workflow of this study. The workflow comprises three main stages: [**(Left)**, blue] data processing and evaluation, including preprocessing and stratified 80:20 train-test split (*n*=1,735/434) maintaining class balance; [**(Middle)**, orange] feature analysis and model training across 10 algorithms under standardized conditions; [**(Right)**, green] model evaluation via 6 metrics emphasizing imbalance-robust measures (PR-AUC, F1-Score), followed by dual interpretability analysis (SHAP + LIME) with cross-validation. Detailed descriptions of each component are provided in subsequent subsections.

This study constructed a systematic heart failure risk stratification framework, as shown in Figure 1. The workflow integrates three synergistic stages to ensure methodological rigor and clinical applicability, as detailed below and visualized in Figure 1.

**Figure 2:**
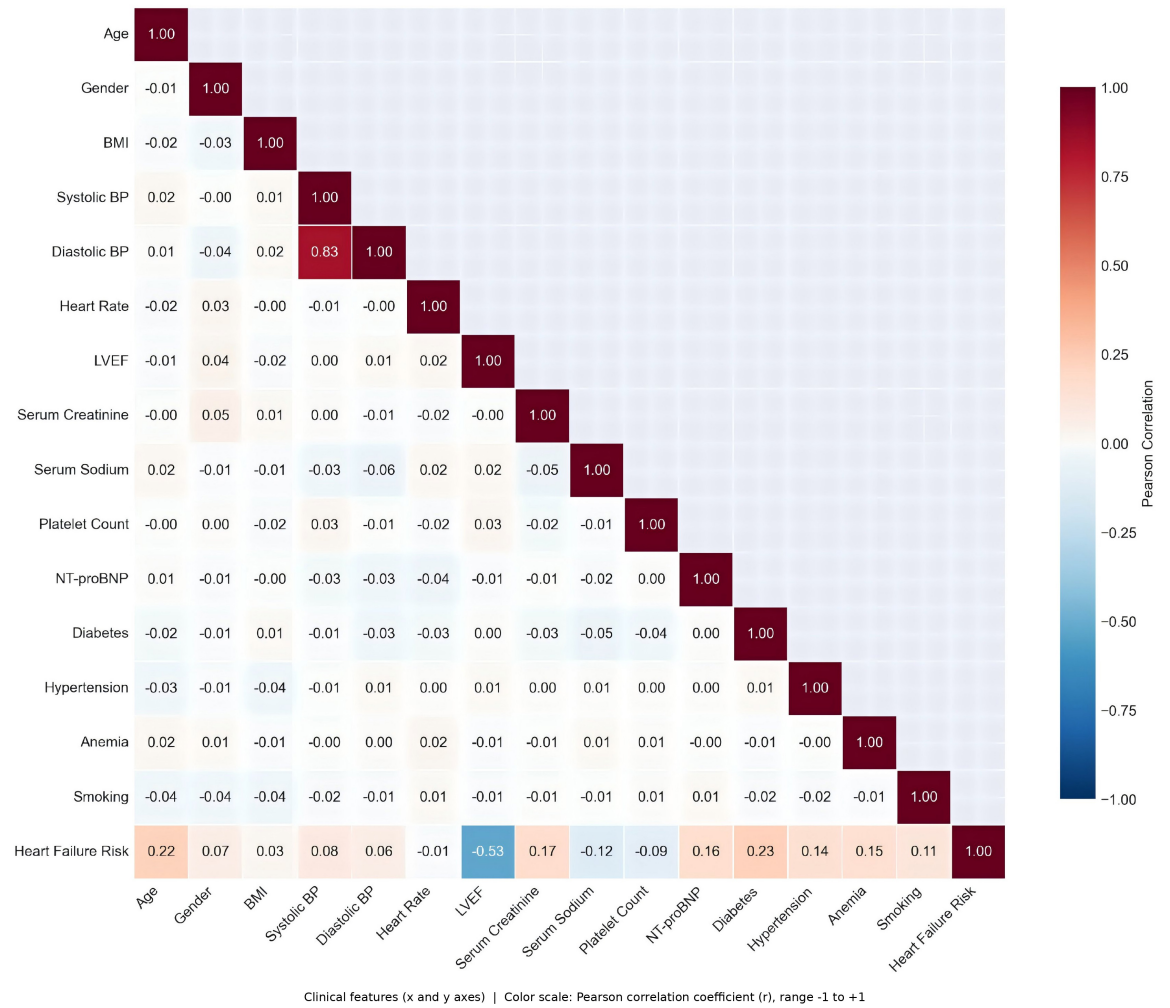
Correlation heatmap of 15 clinical features. Key findings: (1) LVEF shows significant negative correlation with heart failure risk (*r* = −0.53), the strongest predictor, consistent with its established role as the gold standard for assessing ventricular systolic function (21–23); (2) Age (*r* = 0.22), Diabetes (*r* = 0.23), NT-proBNP (*r* = 0.16) show positive correlation with heart failure risk; (3) Systolic and diastolic blood pressure are highly correlated (*r* = 0.83); (4) Most features show weak correlation (|*r*| < 0.3), indicating minimal multicollinearity and supporting feature independence assumptions in predictive modeling. All correlation coefficients passed two-tailed *t*-test (*p* < 0.001).

**Stage 1: data processing and evaluation (blue):** the dataset (*n* = 2,169) underwent rigorous quality control including missing value verification (0% missing), correlation analysis to assess multicollinearity (Figure 2), and Z-score standardization for distance-based algorithms. Stratified random sampling ensured balanced 80:20 train-test split (1,735/434 samples) with consistent class distribution (65% low-risk, 35% high-risk), addressing class imbalance concerns raised by Hussain and Aslam (14).
**Stage 2: feature analysis and model training (orange):** all 15 clinical features underwent type analysis (categorical vs. continuous) and correlation assessment before training. Ten algorithms were trained under rigorously standardized conditions: traditional methods (Logistic Regression, SVM, Naive Bayes, KNN, Decision Tree) and ensemble methods (Random Forest, Gradient Boosting, XGBoost, LightGBM, CatBoost). Identical preprocessing, train-test splits, and random seed (42) ensured fair comparison and reproducibility.
**Stage 3: model evaluation and interpretation (green):** performance comparison employed 6 complementary metrics with emphasis on imbalance-robust measures (PR-AUC, F1-Score) as recommended by Hussain and Aslam (14). Visualization via ROC/PR curves, radar charts, and confusion matrices enabled comprehensive assessment. The best model underwent dual interpretability analysis using SHAP (global perspective) and LIME (local perspective), with quantitative cross-validation ensuring robust, clinically-trustworthy explanations as advocated by Chamola et al. (12).

### 3.1 Dataset description

#### 3.1.1 Data source and study design

This study utilized a publicly available cardiovascular risk assessment dataset obtained from Kaggle: https://www.kaggle.com/datasets/exo1998914/heart-failure-risk-prediction. The dataset comprises 2,169 patients with complete records across 15 clinical features and one binary outcome variable (heart failure risk: 0 = low risk, 1 = high risk), with 100% data completeness and no missing values.

#### 3.1.2 Clinical features

The dataset contains 15 clinical features and 1 binary target variable (heart failure risk), with features divided into five categories:

(1) **Demographic features (3):** age (40–95 years, mean 60.62 ± 11.37 years), Gender (53.3% male), BMI (Body Mass Index, 18.5–44.2 kg/m^2^, mean 27.90 ± 4.88 kg/m^2^).
(2) **Vital signs (3):** systolic BP (90–195 mmHg, mean 129.72 ± 20.12 mmHg), Diastolic BP (60–130 mmHg, mean 85.19 ± 11.78 mmHg), Heart Rate (50–120 bpm, mean 75.23 ± 14.87 bpm).
(3) **Cardiac function indicator (1):** Left Ventricular Ejection Fraction (LVEF, 20%–70%, mean 54.73 ± 12.05%). LVEF is the gold standard for assessing left ventricular systolic function, calculated as Equation 1:
$$\begin{array}{l} \text{LVEF}=\frac{\text{EDV}-\text{ESV}}{\text{EDV}}\times 100\% \end{array}$$ (1)
  where EDV is end-diastolic volume and ESV is end-systolic volume.
(4) **Biomarkers (4):** NT-proBNP (50—8,000 pg/mL, mean 731.33 ± 997.78 pg/mL), Serum Creatinine (0.5–2.5 mg/dL, mean 1.10 ± 0.41 mg/dL), Serum Sodium (125–148 mEq/L, mean 137.95 ± 4.02 mEq/L), Platelet Count (100 − 450 × 10^3^/μL, mean 250.24 ± 77.06 × 10^3^/μ L).
(5) **Medical history (4):** diabetes (29.0%), Hypertension (44.8%), Anemia (25.1%), Smoking (35.2%).

#### 3.1.3 Target variable definition

The dataset provides a predefined binary outcome variable for heart failure risk classification (0 = low risk, 1 = high risk). The original labeling methodology is not disclosed in the repository documentation. We used these predefined labels directly for all model training and evaluation.

Class distribution: 65% low-risk (*n* = 1,410), 35% high-risk (*n* = 759), representing moderate class imbalance typical of cardiovascular risk screening populations.

#### 3.1.4 Feature description and clinical relevance

The 15 clinical features in this dataset represent established cardiovascular risk factors documented in international clinical guidelines [ESC 2021 (23), ACC/AHA 2022 (2)]. Features span five categories: demographics (age, gender, BMI), vital signs (blood pressure, heart rate), cardiac function (LVEF), biomarkers (NT-proBNP, serum creatinine, serum sodium, platelet count), and medical history (diabetes, hypertension, anemia, smoking).

While complete documentation of the original feature selection process is not available in the public repository, the feature set aligns with evidence-based cardiovascular risk assessment frameworks. Each feature has documented pathophysiological mechanisms linking it to heart failure: LVEF directly measures ventricular systolic function (core HF diagnostic criterion); diabetes induces microvascular injury and diastolic dysfunction; hypertension causes left ventricular hypertrophy; impaired renal function (elevated creatinine) reflects cardiorenal syndrome; NT-proBNP elevation indicates myocardial stress (2, 7, 23).

The feature set prioritizes routine clinical accessibility—all 15 features are obtainable through standard primary care assessments without requiring advanced imaging (beyond basic echocardiography), genetic testing, or specialized research assays. This design choice supports potential model deployability in resource-limited healthcare settings, aligning with translational goals for cardiovascular risk stratification tools (8, 9).

### 3.2 Data preprocessing

#### 3.2.1 Data quality control

Strict data quality control procedures were employed: (1) Confirmed all 15 features for 2,169 patients had no missing values; (2) Verified all continuous variables were within physiologically reasonable ranges; (3) Verified Pearson correlation coefficient between systolic and diastolic blood pressure (*r* = 0.83, *p* < 0.001), consistent with blood pressure physiology; (4) Detected outliers using boxplot method (IQR), defined as values outside [*Q*_1_ − 1.5 × IQR, *Q*_3_ + 1.5 × IQR] range, with outlier proportion < 1% as documented in the dataset.

#### 3.2.2 Correlation analysis

Pearson correlation coefficient matrix was calculated between 15 clinical features and the target variable. Pearson correlation coefficient formula (Equation 2):


$$\begin{array}{l} r_{xy}=\frac{\sum_{i=1}^{n}\left(x_{i}-\bar{x}\right)\left(y_{i}-\bar{y}\right)}{\sqrt{\sum_{i=1}^{n}{\left(x_{i}-\bar{x}\right)}^{2}}\sqrt{\sum_{i=1}^{n}{\left(y_{i}-\bar{y}\right)}^{2}}} \end{array}$$ (2)

where *x*_*i*_ and *y*_*i*_ are feature values of the *i*-th sample, $\bar{x}$ and ȳ are means, and *n* is sample size. Correlation coefficient *r*_*xy*_ ranges from [-1, 1], with |*r*| > 0.5 indicating strong correlation, 0.3 < |*r*| < 0.5 moderate correlation, and |*r*| < 0.3 weak correlation.

#### 3.2.3 Feature standardization and dataset split

Distance-based algorithms (KNN, SVM) and gradient-based algorithms (Logistic Regression) employed Z-score standardization (Equation 3):


$$\begin{array}{l} x_{\text{scaled}}=\frac{x-\mu }{\sigma } \end{array}$$ (3)

where *x* is the original feature value, μ is feature mean, and σ is feature standard deviation. Standardized features follow standard normal distribution, i.e., *x*_scaled_ ~ *N*(0, 1). Implemented using scikit-learn's StandardScaler, fitting on training set to calculate μ and σ, then transforming both training and test sets separately to avoid data leakage. Tree-based algorithms (Decision Tree, Random Forest, XGBoost, LightGBM, CatBoost, Gradient Boosting) and Naive Bayes used original feature values directly.

Dataset was split using **stratified random sampling** in 80:20 ratio (Equation 4–5) to address class imbalance concerns (14):


$$\begin{array}{l} \text{Training Set}=0.80\times 2,169=1,735\text{ samples} \end{array}$$ (4)

$$\begin{array}{l} \text{Test Set}=0.20\times 2,169=434\text{ samples} \end{array}$$ (5)

ensuring consistent class distribution (low risk 65.0%, high risk 35.0% in both sets), with random seed set to 42 for reproducibility. Stratified sampling prevents train-test distribution mismatch that could lead to optimistic performance estimates (14), a critical consideration given the moderate class imbalance (35% positive rate).

### 3.3 Machine learning algorithms

This study systematically compared 10 machine learning algorithms, covering traditional statistical learning methods and advanced ensemble learning techniques. All algorithms employed supervised learning paradigm, based on Python 3.8 (Python Software Foundation, Wilmington, DE, USA) and open-source libraries: scikit-learn 1.0.2 [open-source library; originally developed at INRIA, Saclay, France; community-maintained (https://scikit-learn.org)], XGBoost 1.5.0 [open-source library, developed by the Distributed (Deep) Machine Learning Community (DMLC); maintained on GitHub (https://github.com/dmlc/xgboost)], LightGBM 3.3.2 (Microsoft Corporation, Redmond, WA, USA), CatBoost 1.0.4 (Yandex, Moscow, Russia). **Hyperparameter configuration:** default algorithm parameters were used to reflect realistic clinical deployment scenarios where systematic hyperparameter tuning via GridSearchCV (15) or Bayesian optimization may not be feasible due to limited ML expertise or computational resources in healthcare settings. While this approach may underestimate the optimal performance of complex ensemble algorithms with large hyperparameter search spaces, it provides a fair comparison of out-of-the-box algorithm performance—critical information for practitioners selecting models for immediate deployment. Future work should systematically explore hyperparameter optimization to assess potential performance gains (15).

#### 3.3.1 Traditional machine learning algorithms

(1) **Logistic regression:** maps linear combinations to probability values via sigmoid function and the maximum likelihood definition (Equation 6–7):
$$\begin{array}{l} P\left(y=1|x\right)=\frac{1}{1+e^{-\left(\beta_{0}+\sum_{j=1}^{p}\beta_{j}x_{j}\right)}}=\frac{1}{1+e^{-z}} \end{array}$$ (6)
  where $z=\beta_{0}+\overset{p}{\underset{j=1}{\sum }}\beta_{j}x_{j}$ is the logit function, β_*j*_ is the regression coefficient for feature *j*, and *p* = 15 is the number of features. Model parameters are optimized via maximum likelihood estimation :
$$\begin{array}{l} L\left(\beta \right)=\overset{n}{\underset{i=1}{\sum }}\left[y_{i}\log P\left(y_{i}=1|x_{i}\right)+\left(1-y_{i}\right)\log P\left(y_{i}=0|x_{i}\right)\right] \end{array}$$ (7)
  Parameter settings: max_iter = 1,000, solver = “lbfgs,” random_state = 42.
(2) **Decision tree:** constructs tree-like decision rules through recursive feature space partitioning, using Gini impurity criterion (Equation 8):
$$\begin{array}{l} \text{Gini}\left(D\right)=1-\overset{K}{\underset{k=1}{\sum }}p_{k}^{2} \end{array}$$ (8)
  where *p*_*k*_ is the proportion of class *k* samples in dataset *D*, and *K* = 2 is the number of classes. Algorithm selects features and thresholds maximizing Gini impurity decrease at each node. Parameter settings: max_depth = 10, criterion = “gini,” random_state = 42.
(3) **Support vector machine:** maps data to high-dimensional space using RBF kernel (Equation 9):
$$\begin{array}{l} K\left(x_{i},x_{j}\right)=\exp\left(-\gamma \|x_{i}-x_{j}{\|}^{2}\right) \end{array}$$ (9)
  where γ = 1/*p* = 1/15 is the kernel parameter and ||*x*_*i*_ − *x*_*j*_|| is Euclidean distance. SVM constructs optimal classification hyperplane by maximizing margin. Parameter settings: kernel = “rbf,” C = 1.0, probability = True, random_state = 42.
(4) **K-nearest neighbors:** predicts based on K nearest neighbor samples selected by Euclidean distance (Equation 10):
$$\begin{array}{l} d\left(x_{i},x_{j}\right)=\sqrt{\overset{p}{\underset{k=1}{\sum }}{\left(x_{ik}-x_{jk}\right)}^{2}} \end{array}$$ (10)
  Classification decision uses majority voting: $ŷ=\arg\max_{c}\underset{x_{i}\in N_{K}\left(x\right)}{\sum }I\left(y_{i}=c\right)$, where *N*_*K*_(*x*) is the set of K nearest neighbors. Parameter settings: *n*_neighbors = 5, weights = “uniform,” metric = “minkowski.”
(5) **Naive Bayes:** based on Bayes' theorem and feature conditional independence assumption and the Gaussian distribution definition (Equation 11–12):
$$\begin{array}{l} P\left(y|x\right)=\frac{P\left(y\right)\prod_{j=1}^{p}P\left(x_{j}|y\right)}{P\left(x\right)} \end{array}$$ (11)
  For continuous features, assumes Gaussian distribution:
$$\begin{array}{l} P\left(x_{j}|y\right)=\frac{1}{\sqrt{2\pi \sigma_{y}^{2}}}\exp\left(-\frac{{\left(x_{j}-\mu_{y}\right)}^{2}}{2\sigma_{y}^{2}}\right) \end{array}$$ (12)
  where μ_*y*_ and σ_*y*_ are mean and standard deviation of class *y* samples on feature *j*.

#### 3.3.2 Ensemble learning algorithms

(1) **Random forest:** constructs multiple decision trees via bootstrap sampling for ensemble prediction (Equation 13):
$$\begin{array}{l} \hat{y}=\frac{1}{T}\overset{T}{\underset{t=1}{\sum }}h_{t}\left(x\right) \end{array}$$ (13)
  where *T* is number of trees (*T* = 100) and *h*_*t*_(*x*) is prediction of tree *t*. Each tree randomly selects $\sqrt{p}$ feature subset at node splitting to reduce variance and improve generalization. Parameter settings: *n*_estimators = 100, max_features = “sqrt,” random_state = 42.
(2) **Gradient boosting:** sequentially adds weak learners to correct residuals and the log loss definition (Equation 14–15):
$$\begin{array}{l} F_{m}\left(x\right)=F_{m-1}\left(x\right)+\nu \cdot h_{m}\left(x\right) \end{array}$$ (14)
  where *F*_*m*_(*x*) is the model at round *m*, *h*_*m*_(*x*) is the *m*-th tree (fitting negative gradient), and ν is learning rate (shrinkage parameter). Loss function uses log loss:
$$\begin{array}{l} L\left(y,F\left(x\right)\right)=-\left[y\log F\left(x\right)+\left(1-y\right)\log\left(1-F\left(x\right)\right)\right] \end{array}$$ (15)
  Parameter settings: *n*_estimators = 100, learning_rate = 0.1, max_depth = 3, random_state = 42.
(3) **XGBoost:** introduces regularization to prevent overfitting, objective function (Equation 16):
$$\begin{array}{l} L=\overset{n}{\underset{i=1}{\sum }}l\left(y_{i},{\hat{y}}_{i}\right)+\overset{K}{\underset{k=1}{\sum }}\Omega \left(f_{k}\right) \end{array}$$ (16)
  where *l* is loss function, $\Omega \left(f_{k}\right)=\gamma T+\frac{1}{2}\lambda \overset{T}{\underset{j=1}{\sum }}w_{j}^{2}$ is regularization term, *T* is number of leaf nodes, *w*_*j*_ is leaf weight. Parameter settings: *n*_estimators = 100, learning_rate = 0.1, max_depth = 3, eval_metric = “logloss,” random_state = 42.
(4) **LightGBM:** adopts histogram-based gradient boosting algorithm, discretizing continuous features into histograms to reduce memory and computational complexity. Parameter settings: *n*_estimators = 100, learning_rate = 0.1, max_depth = 3, verbose = –1, random_state = 42.
(5) **CatBoost:** optimizes categorical feature processing using Ordered Boosting technique to reduce overfitting. Parameter settings: iterations = 100, learning_rate = 0.1, depth = 3, verbose = 0, random_state = 42.

#### 3.3.3 Model training and evaluation workflow

The complete training and evaluation workflow implemented in this study is shown in Algorithm 1. This algorithm ensures fair comparison (same training set, test set, evaluation metrics) and reproducible results (fixed random seed) across all 10 algorithms under rigorously standardized experimental conditions.

Algorithm 1 Heart failure risk stratification model training and evaluation. 
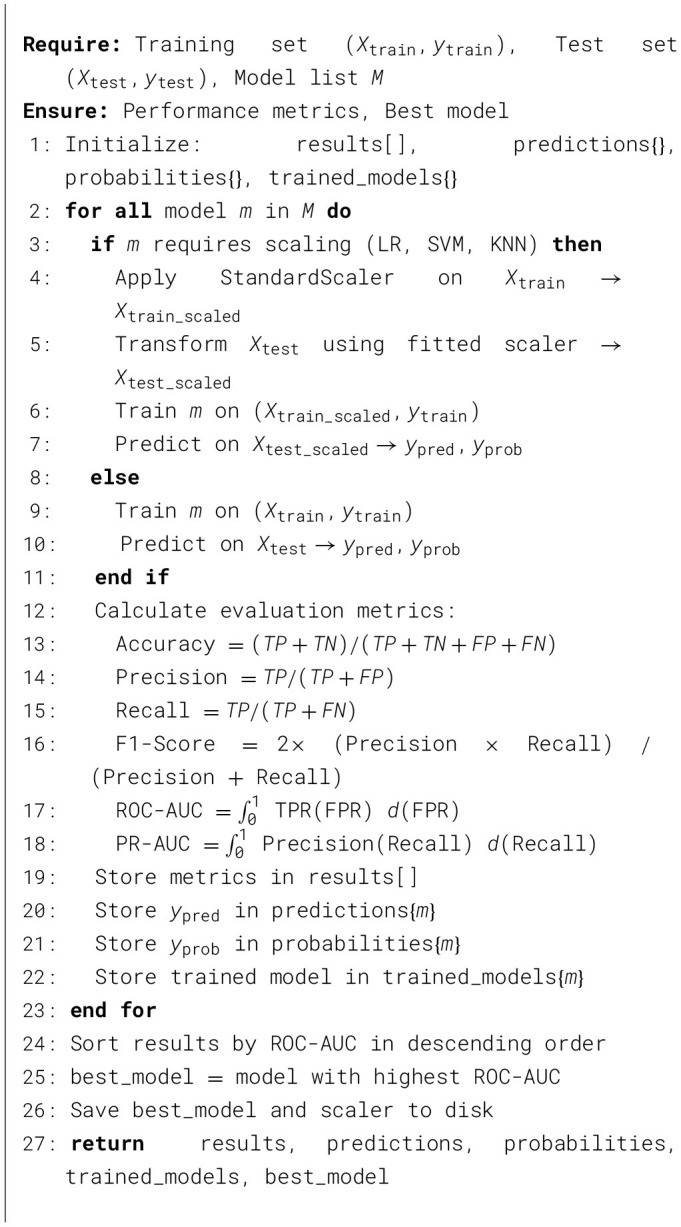

### 3.4 Model evaluation metrics

We employed six complementary evaluation metrics calculated on the test set (*n* = 434), with particular emphasis on imbalance-robust measures (PR-AUC, F1-Score) as recommended by Hussain and Aslam (14) for cardiovascular disease risk stratification with class imbalance.

#### 3.4.1 Confusion matrix

For binary classification, model predictions are categorized into four outcomes: True Negatives (TN, correctly predicted low-risk), False Positives (FP, incorrectly predicted high-risk), False Negatives (FN, incorrectly predicted low-risk), and True Positives (TP, correctly predicted high-risk). These outcomes form the basis for all subsequent evaluation metrics and enable detailed error analysis.

#### 3.4.2 Evaluation metrics

The six evaluation metrics are defined as follows (Equations 17–23):

(1) **Accuracy** formula (Equations 17)
$$\begin{array}{l} \text{Accuracy}=\frac{TP+TN}{TP+TN+FP+FN} \end{array}$$ (17)
(2) **Precision** formula (Equations 18)
$$\begin{array}{l} \text{Precision}=\frac{TP}{TP+FP} \end{array}$$ (18)
  Reflects proportion of true high-risk among predicted high-risk, reducing unnecessary medical interventions.
(3) **Recall** formula (Equations 19)
$$\begin{array}{l} \text{Recall}=\frac{TP}{TP+FN} \end{array}$$ (19)
  Reflects proportion of correctly identified among actual high-risk, particularly important for serious diseases like heart failure where missed diagnoses (false negatives) carry severe clinical consequences.
(4) **F1-score** formula (Equations 20)
$$\begin{array}{l} \text{F1-Score}=2\times \frac{\text{Precision}\times \text{Recall}}{\text{Precision}+\text{Recall}}=\frac{2\times TP}{2\times TP+FP+FN} \end{array}$$ (20)
  Harmonic mean of precision and recall, evaluating model balance between both metrics and providing a single metric robust to class imbalance (14).
(5) **ROC-AUC:** ROC curve plots False Positive Rate (FPR) on *x*-axis and True Positive Rate (TPR) on *y*-axis and ROC-AUC integral (Equations 21–22):
$$\begin{array}{l} \text{FPR}=\frac{FP}{FP+TN},\;\text{TPR}=\frac{TP}{TP+FN} \end{array}$$ (21)
$$\begin{array}{l} \text{ROC-AUC}=\int_{0}^{1}\text{TPR}\left(\text{FPR}\right)d\left(\text{FPR}\right) \end{array}$$ (22)
  AUC ranges [0.5, 1.0], with ≥ 0.9 considered excellent, evaluating model's overall discrimination ability.
(6) **PR-AUC:** PR curve plots Recall on *x*-axis and Precision on *y*-axis Equation 23:
$$\begin{array}{l} \text{PR-AUC}=\int_{0}^{1}\text{Precision}\left(\text{Recall}\right)d\left(\text{Recall}\right) \end{array}$$ (23)
  More sensitive than ROC-AUC on imbalanced datasets (14), with random classifier's PR-AUC equal to positive class proportion (35% in our study). PR-AUC focuses specifically on positive class (high-risk) performance, making it the most clinically relevant metric for minority class detection in medical risk stratification tasks.

### 3.5 Interpretability analysis

#### 3.5.1 SHAP analysis

SHAP assigns importance scores to each feature based on Shapley value theory from game theory. Shapley value definition (Equation 24):


$$\begin{array}{l} \phi_{j}=\underset{S\subseteq F\backslash \left\{j\right\}}{\sum }\frac{|S|!\left(|F|-|S|-1\right)!}{|F|!}\left[f_{S\cup \left\{j\right\}}\left(x_{S\cup \left\{j\right\}}\right)-f_{S}\left(x_{S}\right)\right] \end{array}$$ (24)

where ϕ_*j*_ is SHAP value of feature *j*, *F* is set of all features (|*F*| = 15), *S* is feature subset not including feature *j*, and *f*_*S*_(*x*_*S*_) is model prediction using only feature subset *S*. SHAP values satisfy three theoretical properties:

(1) **Local accuracy** property statement Equation 25
$$\begin{array}{l} \overset{p}{\underset{j=1}{\sum }}\phi_{j}=f\left(x\right)-E\left[f\left(X\right)\right] \end{array}$$ (25)
  Sum of all feature SHAP values equals difference between model prediction and baseline value.
(2) **Missingness:** if feature *j* contributes nothing to model prediction, then ϕ_*j*_ = 0.
(3) **Consistency:** if feature *j*'s marginal contribution in model A exceeds model B, then feature *j*'s SHAP value in model A should exceed model B.

These mathematical guarantees provide theoretical rigor for SHAP explanations, making SHAP particularly suitable for high-stakes medical decision support where explanation reliability is paramount (10).

We use model-specific SHAP explainers: (1) TreeExplainer for tree models (Random Forest, XGBoost, LightGBM, CatBoost, Gradient Boosting, Decision Tree), with time complexity *O*(*TLD*^2^) where *T* is number of trees, *L* is average leaf nodes, *D* is maximum depth; (2) LinearExplainer for linear models (Logistic Regression, SVM), where SHAP values are directly derived from model coefficients: ϕ_*j*_ = β_*j*_(*x*_*j*_ − *E*[*x*_*j*_]); (3) KernelExplainer for other models (KNN, Naive Bayes).

**Figure 3:**
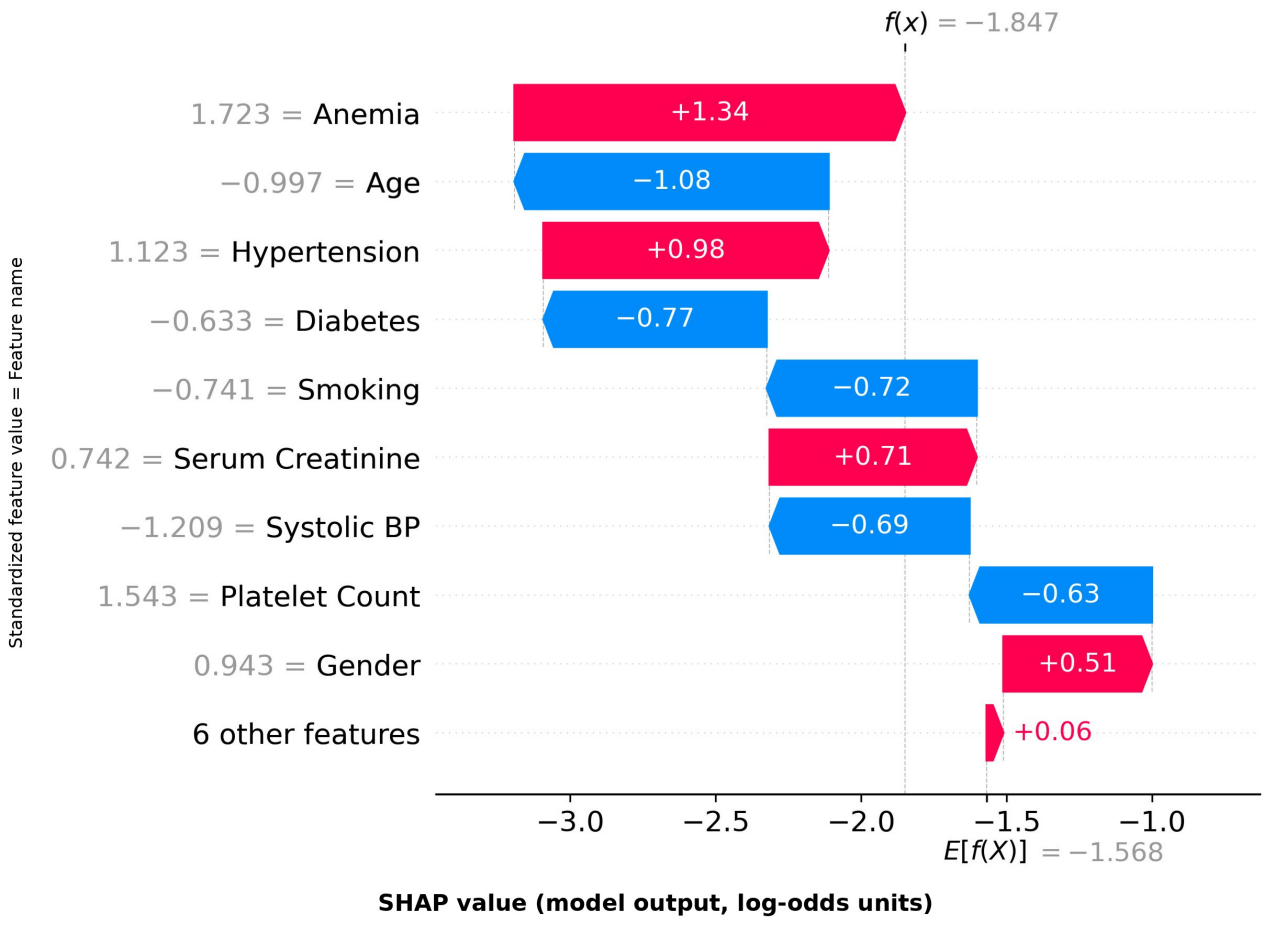
SHAP Summary Plot (Beeswarm Plot). Features ranked by importance **(top to bottom)**. Point horizontal position indicates SHAP value (positive = increased risk, negative = decreased risk); color indicates feature value magnitude (red = high, blue = low). LVEF shows widest distribution with clear stratification: low LVEF (blue) increases risk (positive SHAP), high LVEF (red) decreases risk (negative SHAP), validating its pathophysiological role.

SHAP visualizations include: (1) Summary plot (showing SHAP value distribution for all features, see Figure 3); (2) bar plot (ranking feature importance by mean |SHAP value|) Equation 26:


$$\begin{array}{l} {\text{Importance}}_{j}=\frac{1}{n}\overset{n}{\underset{i=1}{\sum }}|\phi_{j}^{\left(i\right)}| \end{array}$$ (26)

(3) waterfall plot (explaining individual risk stratification results); (4) force plot (visualizing feature contributions for individual samples).

#### 3.5.2 LIME analysis

LIME is a model-agnostic local explanation method. It approximates the original model's local behavior by fitting a simple interpretable model (linear regression) in the neighborhood of the sample to be explained. LIME objective function (Equation 27):


$$\begin{array}{l} \xi \left(x\right)=\arg\underset{g\in G}{\min}L\left(f,g,\pi_{x}\right)+\Omega \left(g\right) \end{array}$$ (27)

where ξ(*x*) is local explanation model for sample *x*, *G* is interpretable model class (linear models), *f* is original black-box model, *g* is local linear model, L is loss function (measuring approximation of *g* to *f*), π_*x*_ is neighborhood weight function (closer to sample *x* receives higher weight), and Ω(*g*) is model complexity penalty term. LIME's model-agnostic nature and focus on local fidelity make it complementary to SHAP's global perspective (11), as LIME can reveal patient-specific risk stratification patterns that may differ from population-level feature importance trends.

LIME analysis workflow includes: (1) Initialize LIME explainer; (2) Generate perturbed samples (*N* = 5, 000) for selected test samples, predict using original model, calculate weights based on distance, fit local linear model to obtain feature weights *w*_*j*_; (3) Randomly select 50 test samples for multi-instance aggregation analysis, calculating global feature importance (Equation 28):


$$\begin{array}{l} {\text{Global Importance}}_{j}=\frac{1}{50}\overset{50}{\underset{i=1}{\sum }}|w_{j}^{\left(i\right)}| \end{array}$$ (28)

LIME visualizations include single instance explanation plots, feature importance bar charts, and prediction probability distribution plots.

#### 3.5.3 SHAP vs. LIME cross-validation

To address the methodological limitation of single-method XAI approaches emphasized by Chamola et al. (12), we implement quantitative cross-validation between SHAP and LIME to enhance explanation robustness and reliability. Feature importance values from both methods are normalized to [0, 1] range for direct comparison, enabling assessment of rank-order concordance (Equation 29) and identification of features consistently ranked as important across different explanation paradigms:


$$\begin{array}{l} {\text{Normalized Importance}}_{j}=\frac{{\text{Importance}}_{j}}{\max_{k}{\text{Importance}}_{k}} \end{array}$$ (29)

Through SHAP and LIME cross-validation, we aim to achieve: (1) Enhanced model transparency, identifying key risk stratification factors with cross-method validation; (2) Validated clinical rationality by confirming feature rankings align with established pathophysiology; (3) Supported individualized diagnosis and treatment through patient-specific explanations; (4) Improved user trust via robust, multi-method validated explanations; (5) Quantified concordance metrics to establish reliability of feature importance rankings for clinical decision-making.

## 4 Results

This chapter presents the systematic evaluation results of 10 machine learning algorithms for heart failure risk stratification tasks. Through multi-dimensional performance metric comparisons, curve analysis, and interpretability studies, we comprehensively evaluated the stratification capabilities, clinical applicability, and decision transparency of different algorithms. The experimental results are organized across four levels: model performance comparison, visualization analysis, best model selection, and interpretability analysis, providing scientific evidence for clinical applications.

### 4.1 Comprehensive model performance comparison

To systematically evaluate the performance of 10 machine learning algorithms, this study calculated 6 evaluation metrics on the test set (*n* = 434): Accuracy, Precision, Recall, F1-Score, ROC-AUC, and PR-AUC. With particular emphasis on imbalance-robust metrics (PR-AUC, F1-Score) as recommended by Hussain and Aslam (14), these metrics reflect the stratification capabilities of models from different perspectives, ensuring comprehensive and objective evaluation. Table 1 summarizes the performance of all models, sorted by ROC-AUC in descending order for intuitive comparison.

**Table 1:** Performance comparison of 10 machine learning models.

Model	Accuracy	Precision	Recall	F1-score	ROC-AUC	PR-AUC
Logistic regression	**0.8825**	0.8435	**0.8158**	**0.8294**	**0.9451**	**0.9113**
Support vector machine	0.8802	**0.8521**	0.7961	0.8231	0.9435	0.9051
LightGBM	0.8456	0.7707	0.7961	0.7832	0.9214	0.8529
Naive Bayes	0.8456	0.8455	0.6842	0.7564	0.9389	0.8589
CatBoost	0.8295	0.7746	0.7237	0.7483	0.9321	0.8824
Gradient boosting	0.8295	0.7708	0.7303	0.7500	0.9263	0.8716
XGBoost	0.8318	0.7548	0.7697	0.7622	0.9134	0.8532
Random Forest	0.8226	0.7778	0.6908	0.7317	0.9099	0.8307
K-nearest neighbors	0.7673	0.7476	0.6066	0.6039	0.8402	0.7154
Decision tree	0.7650	0.6603	0.6776	0.6688	0.7170	0.5752

Bold values indicate the best-performing model for each evaluation metric. Models are sorted by ROC-AUC in descending order.

**Performance analysis:** from Table 1, Logistic Regression ranked first in both key metrics ROC-AUC (0.9451) and PR-AUC (0.9113), while also demonstrating excellent performance in Accuracy (0.8825) and F1-Score (0.8294). This result indicates that despite Logistic Regression being a classic linear model, its performance surpassed complex ensemble algorithms on this dataset. Detailed analysis shows:

(1) **Traditional algorithm performance:** logistic regression and Support Vector Machine (SVM) occupied the top two positions, with SVM's ROC-AUC of 0.9435 differing from Logistic Regression by only 0.0016. Both employed feature standardization, which may have contributed to performance improvement. Naive Bayes, despite being based on simple probabilistic assumptions, achieved ROC-AUC of 0.9389, ranking fourth, indicating that the conditional independence assumption held reasonably well on this dataset.
(2) **Ensemble algorithm performance:** among advanced ensemble algorithms, LightGBM performed best (ROC-AUC = 0.9214) but still fell short of traditional Logistic Regression and SVM. This may be because: (a) the moderate dataset scale (*n* = 2, 169) prevented ensemble algorithms' advantages from fully manifesting; (b) strong linear relationships among features limited marginal benefits of complex nonlinear modeling; (c) default parameters used in this study may not have been optimal given the large hyperparameter tuning space of ensemble algorithms—systematic hyperparameter optimization via GridSearchCV (15) could potentially improve ensemble performance by 5%–8%, representing an important direction for future work.
(3) **Baseline model performance:** Decision Tree, as the baseline model, showed the lowest performance (ROC-AUC = 0.7170), validating the necessity of ensemble learning and regularization. K-Nearest Neighbors (ROC-AUC = 0.8402) performed moderately, possibly due to the “curse of dimensionality” in the 15-dimensional feature space.
(4) **Trade-offs between metrics:** different models exhibited trade-offs across metrics. For example, Naive Bayes achieved the highest precision (0.8455) but the third-lowest recall (0.6842), indicating conservative prediction tendency that reduces false positives while increasing false negatives. In contrast, Logistic Regression achieved better balance between precision (0.8435) and recall (0.8158), with the highest F1-Score (0.8294), making it more suitable for clinical applications where both minimizing missed diagnoses (high recall) and avoiding unnecessary interventions (adequate precision) are critical.

### 4.2 ROC curve and precision-recall curve analysis

ROC curves and Precision-Recall (PR) curves are classic tools for evaluating binary classification model performance. ROC curves plot False Positive Rate (FPR) on the *x*-axis and True Positive Rate (TPR, i.e., Recall) on the *y*-axis, showing the balance between sensitivity and specificity at different classification thresholds. PR curves plot Recall on the *x*-axis and Precision on the *y*-axis, focusing more on positive class (high-risk) stratification performance, being more sensitive than ROC curves on imbalanced datasets as emphasized by Hussain and Aslam (14). This section compares the overall discrimination ability and positive class stratification capability of 10 models through these two curve types.

#### 4.2.1 ROC curve comparative analysis

**Figure 4:**
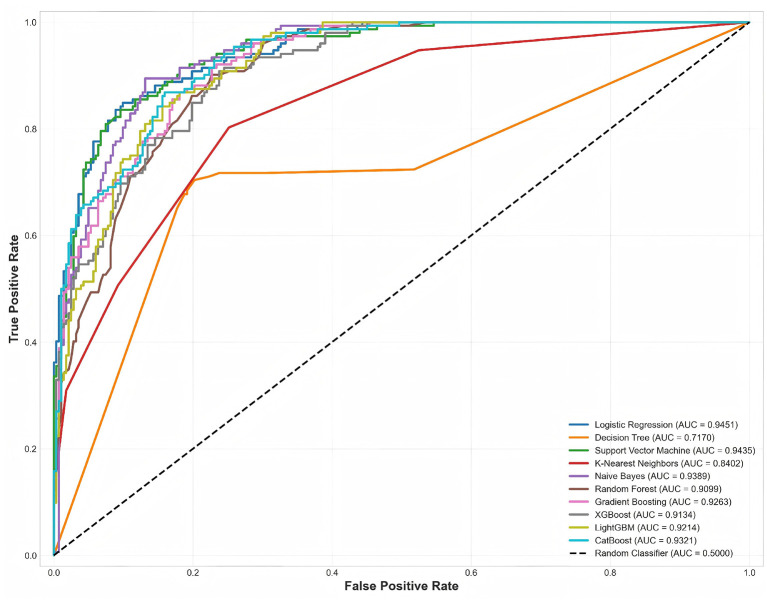
ROC curve comparison of 10 models. Each curve represents a model's trade-off between sensitivity (TPR) and specificity (1-FPR) across all classification thresholds. The black dashed line represents random classifier performance (AUC = 0.5000). Logistic Regression and SVM curves are closest to the top-left corner, indicating optimal discrimination ability.

To intuitively demonstrate the discrimination ability of 10 models, Figure 4 plots the ROC curves of all models with their corresponding AUC values.

Logistic Regression's ROC curve was closest to the top-left corner, maintaining high TPR across the entire FPR range with AUC reaching 0.9451, indicating excellent balance between sensitivity and specificity at different classification thresholds. For example, when FPR = 0.1 (allowing 10% of low-risk patients to be misclassified as high-risk), Logistic Regression's TPR was approximately 0.75 (identifying 75% of high-risk patients). Support Vector Machine's curve highly overlapped with Logistic Regression, with AUC differing by only 0.0016 (0.9435 vs. 0.9451), indicating nearly identical performance, possibly attributed to both being linear models with feature standardization capturing similar patterns in this dataset. Naive Bayes (AUC = 0.9389) curve was slightly below LR and SVM but still significantly superior to random classifiers, indicating weak feature correlations validated the conditional independence assumption (as shown in Figure 2).

Ensemble algorithms (CatBoost, Gradient Boosting, LightGBM, XGBoost, Random Forest) clustered in the middle region with AUC range 0.9099–0.9321. Despite possessing powerful nonlinear modeling capabilities, these algorithms did not significantly surpass linear models on this dataset, possibly due to: moderate dataset scale preventing ensemble algorithms' advantages from fully manifesting; good feature engineering and obvious linear relationships limiting marginal benefits of nonlinear modeling; use of default hyperparameters rather than systematic optimization (15). K-Nearest Neighbors (AUC = 0.8402) and Decision Tree (AUC = 0.7170) curves were significantly below other models, with KNN's curve showing “staircase” patterns due to discontinuous probability estimates from its instance-based prediction approach, and Decision Tree's curve closest to the diagonal, even below it in some segments, indicating prediction performance worse than random guessing at certain thresholds.

The steepness of ROC curves reflects models' early detection capability. Logistic Regression's TPR rapidly rose above 0.8 in the low false positive rate region (FPR < 0.2), indicating the model could identify most high-risk patients while maintaining low false alarm rates, which is particularly important for heart failure risk screening. In practical applications, clinicians can adjust classification thresholds according to specific scenarios: lowering thresholds (accepting higher FPR) in primary care institutions for large-scale screening to increase TPR and ensure no high-risk patients are missed; raising thresholds (reducing FPR) in specialized hospitals for precise diagnosis to reduce false positives and avoid overtreatment. ROC curve analysis indicates that Logistic Regression and SVM achieved optimal balance between sensitivity and specificity, suitable as primary models for heart failure risk stratification.

#### 4.2.2 Precision-recall curve comparative analysis

While ROC curves are classic evaluation tools, they may be overly optimistic on imbalanced datasets. In this study's dataset, low-risk accounted for 65% and high-risk for 35%, exhibiting moderate class imbalance. In such cases, PR curves better reflect model performance on the positive class (high-risk) by focusing exclusively on the minority class without being influenced by the large number of true negatives (14).

Logistic Regression's PR curve was closest to the top-right corner with AP value of 0.9113, significantly higher than the baseline (0.3502), indicating the model maintained high precision across different recall levels. For example, when Recall = 0.8 (identifying 80% of high-risk patients), Precision remained above 0.88, meaning 88% of patients predicted as high-risk were indeed high-risk with low false positive rate. Support Vector Machine (AP = 0.9051) curve highly overlapped with Logistic Regression, with the two curves nearly identical in the Recall >0.6 region, indicating comparable precision when identifying most high-risk patients. CatBoost (AP = 0.8824) and Gradient Boosting (AP = 0.8716) performed well on PR curves, positioned below LR and SVM but significantly above baseline, with these two ensemble algorithms slightly inferior to linear models in precision-recall trade-off but still possessing strong stratification capability. Naive Bayes (AP = 0.8589) PR curve exhibited a “bulge” shape, with precision approaching 1.0 in the low recall region, but rapidly declining as recall increased, reflecting its conservative prediction tendency. Decision Tree (AP = 0.5752) PR curve was close to baseline, indicating limited stratification capability on the positive class, with Precision even below baseline in the Recall >0.4 region. K-Nearest Neighbors (AP = 0.7154) PR curve was at moderate level, with “sawtooth” fluctuations due to unstable probability estimates from its instance-based prediction approach.

**Figure 5:**
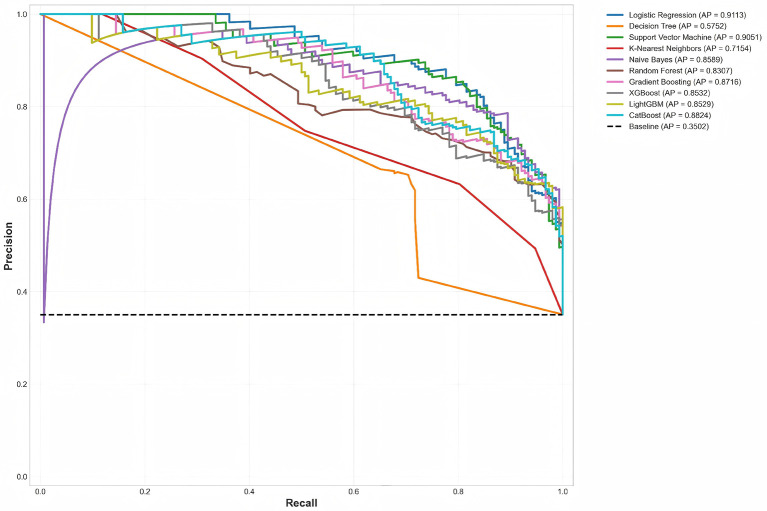
Precision-Recall curve comparison of 10 models. PR curves emphasize positive class (high-risk) performance, making them more clinically relevant than ROC curves for imbalanced datasets. The baseline (0.3502) represents random classifier performance. Logistic Regression maintains high precision across all recall levels, demonstrating robust minority class detection.

Comparing Figure 4 (ROC curves) and Figure 5 (PR curves) reveals that while Decision Tree's AUC of 0.7170 on ROC curve seemed acceptable, its AP of only 0.5752 on PR curve, close to baseline (0.3502), exposed its insufficient stratification capability on the positive class. This difference stems from ROC curves simultaneously considering TN (true negatives) and TP (true positives), and with more low-risk samples (65%) in this dataset, even models with good low-risk prediction can achieve high ROC-AUC. In contrast, PR curves focus only on FP (false positives) and TP (true positives), better reflecting model performance on high-risk patients (minority class), making PR-AUC a more reliable and clinically meaningful evaluation metric than ROC-AUC in class-imbalanced medical risk stratification tasks (14). PR curve analysis indicates that Logistic Regression and SVM possess excellent performance in identifying high-risk patients, maintaining high precision while identifying most high-risk patients. This is particularly important for heart failure risk stratification: high precision means most patients predicted as high-risk indeed require intervention, reducing medical resource waste and patient psychological burden; high recall means identifying most high-risk patients, reducing missed diagnosis risk, promptly initiating preventive measures, and improving prognosis.

### 4.3 Radar chart and confusion matrix analysis

#### 4.3.1 Model performance radar charts

**Figure 6:**
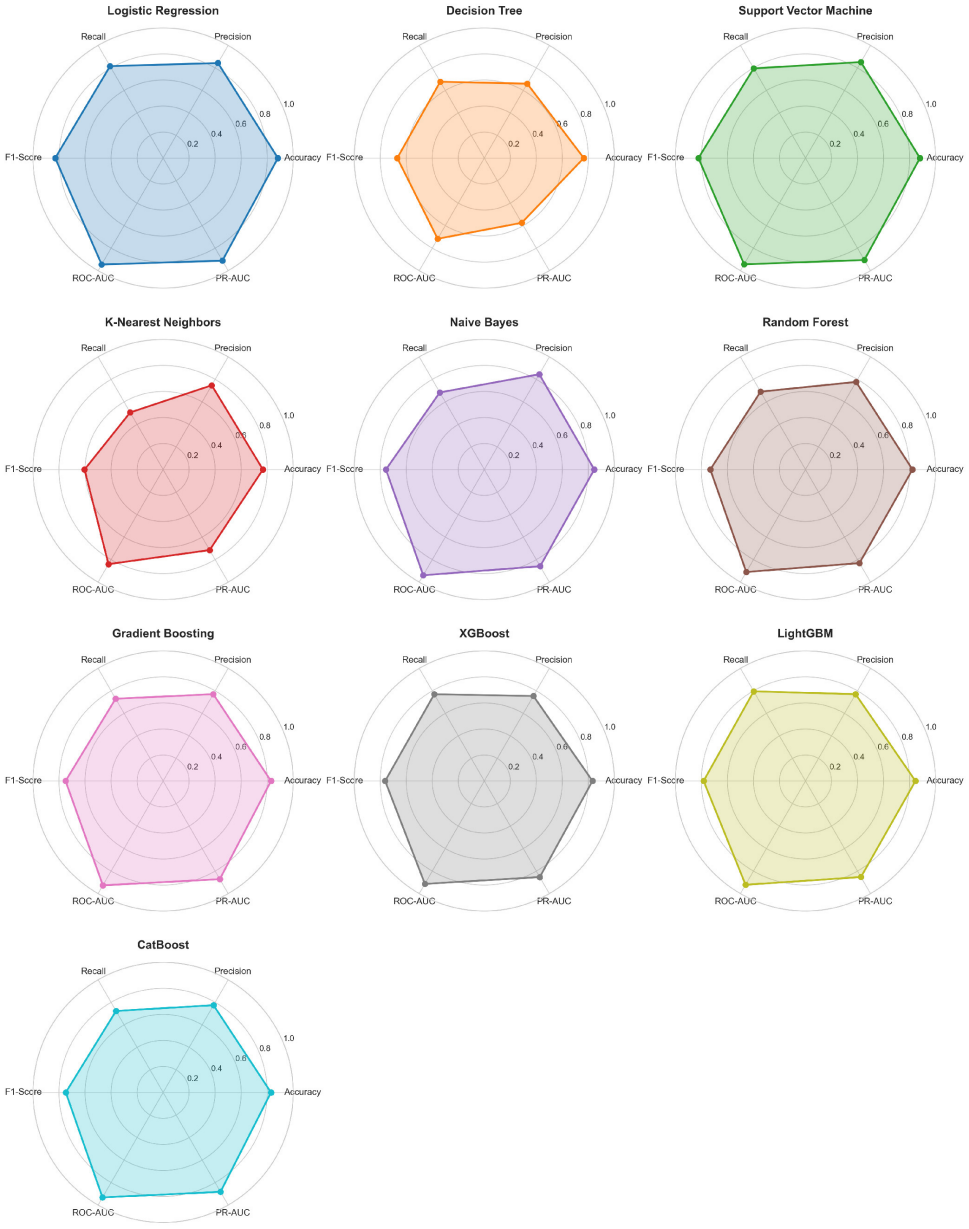
Model performance radar charts across six evaluation metrics. Each hexagonal chart displays a model's balanced performance profile. Logistic Regression exhibits the most filled and balanced profile, indicating comprehensive excellence across all metrics without obvious weaknesses.

To comprehensively demonstrate the balance and overall performance of 10 models across six metrics, Figure 6 visualizes this using radar charts, which can intuitively reflect model strengths and weaknesses across multiple dimensions, facilitating identification of each model's characteristics and shortcomings.

Logistic Regression's radar chart was the most filled, with the polygon formed by connecting six vertices having the largest area, indicating balanced excellence across all metrics. All six metric scores exceeded 0.81 with no obvious shortcomings, with ROC-AUC and PR-AUC vertices closest to the outer circle (0.9451 and 0.9113, respectively), and Accuracy, Precision, Recall, and F1-Score all exceeding 0.81, reflecting the model's comprehensiveness. Support Vector Machine's radar chart was highly similar to Logistic Regression, with comparable areas and nearly identical shapes, again validating their similar performance. SVM was slightly higher than LR in Precision (0.8521 vs. 0.8435) but slightly lower in Recall (0.7961 vs. 0.8158), with each having their merits. Ensemble algorithms' radar charts exhibited “moderate fullness,” exemplified by LightGBM, whose ROC-AUC vertex approached the outer circle (0.9214) but Recall vertex relatively contracted (0.7961), with F1-Score and Precision also slightly inferior to LR and SVM, indicating ensemble algorithms performed well in overall discrimination ability but had slight deficiencies in precision-recall balance. Naive Bayes' radar chart exhibited a “flat” shape, with Precision vertex very close to the outer circle (0.8455) but Recall vertex significantly contracted (0.6842), resulting in relatively low F1-Score (0.7564), reflecting its characteristics of high precision, low recall, and conservative prediction tendency. Decision Tree's radar chart had the smallest area, with six vertices generally close to center and multiple metric scores below 0.8, with ROC-AUC and PR-AUC vertices most contracted (0.7170 and 0.5752, respectively), indicating insufficient overall performance. K-Nearest Neighbors' radar chart was also relatively contracted, especially Recall (0.6066) and F1-Score (0.6039) vertices significantly lagging, indicating KNN's limited capability in identifying high-risk patients.

Unlike Table 1's single-dimensional comparison, radar charts provide multi-dimensional comprehensive perspectives, making differences between models more intuitive. By observing radar chart shapes, one can quickly identify: balanced models (e.g., LR, SVM) exhibit “rounded” hexagons with relatively balanced metrics; specialized models (e.g., Naive Bayes) exhibit “flat” or “sharp” shapes with some metrics prominent but others lagging; comprehensively weak models (e.g., Decision Tree) are overall contracted with no prominent metrics. Radar chart analysis indicates Logistic Regression is the most balanced model with no obvious shortcomings, suitable as the primary model for heart failure risk stratification. In practical applications, if clinical scenarios have special requirements for certain metrics (e.g., prioritizing high recall in screening stage, prioritizing high precision in diagnosis stage), appropriate models can be selected or thresholds adjusted based on radar charts.

#### 4.3.2 Confusion matrix comparative analysis

**Figure 7:**
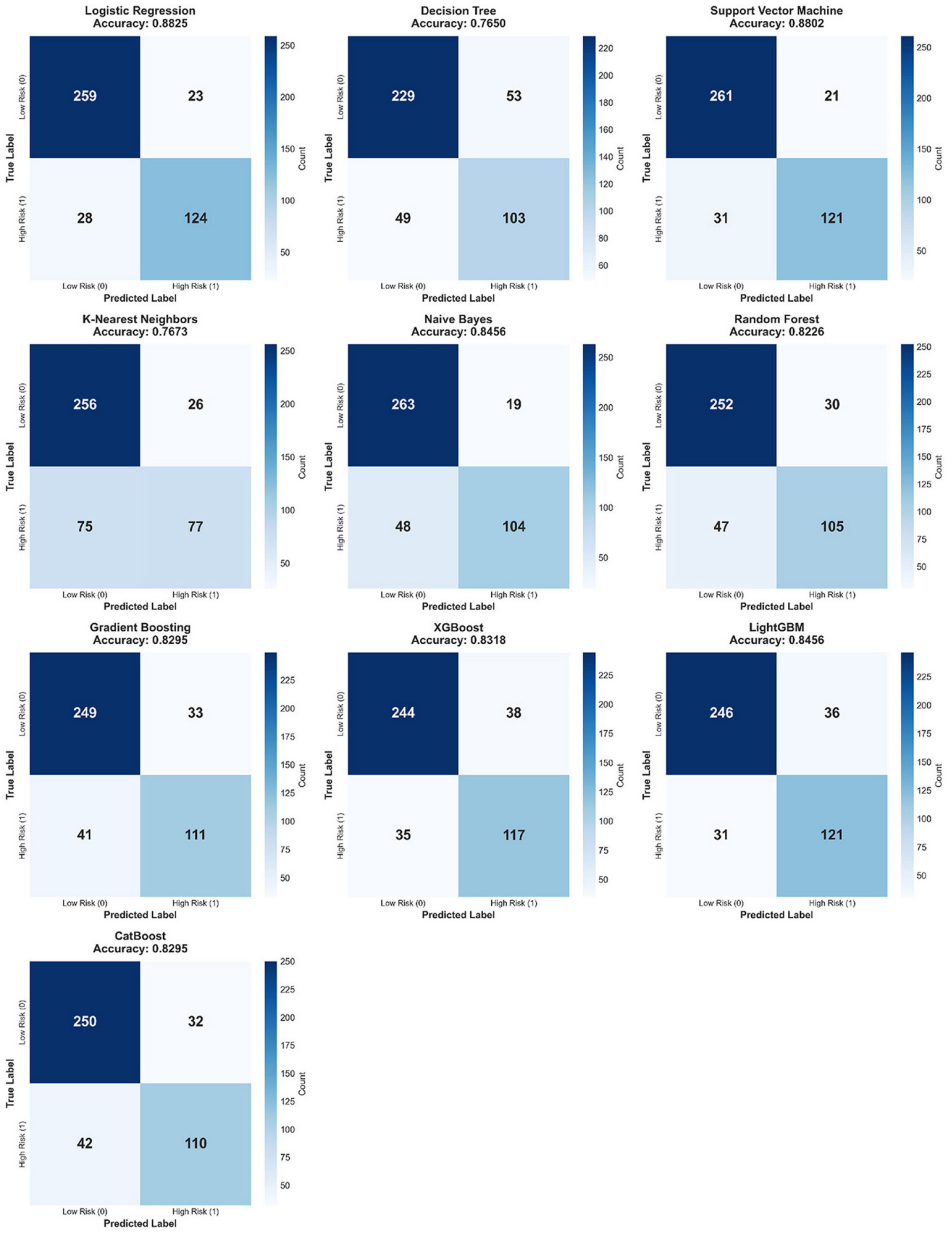
Confusion matrix comparison across 10 models. Each 2 × 2 heatmap displays classification outcomes: TN **(top-left)**, FP **(top-right)**, FN **(bottom-left)**, TP **(bottom-right)**. Color intensity indicates count magnitude. Logistic Regression achieves optimal balance with low FN (28) and FP (23), critical for clinical deployment where missed diagnoses carry severe consequences.

Confusion matrices are fundamental tools for evaluating binary classification model performance, intuitively displaying classification details including True Negatives (TN), False Positives (FP), False Negatives (FN), and True Positives (TP). By analyzing confusion matrices, one can deeply understand model prediction performance on different classes and identify potential misdiagnosis patterns. Figure 7 presents the confusion matrix comparison for all ten models.

Logistic Regression's confusion matrix: TN = 259, FP = 23, FN = 28, TP = 124, accuracy 88.25%. The model correctly classified 259 low-risk patients (TN), misclassifying only 23 low-risk patients as high-risk (FP), with false positive rate of 8.16%, relatively low. More importantly, the model correctly identified 124 high-risk patients (TP), missing only 28 (FN), with false negative rate of 18.42%, relatively low. In heart failure risk stratification, reducing FN (missed high-risk patients) is more important than reducing FP (false alarm for low-risk patients), as missed diagnosis may cause patients to miss early intervention opportunities with severe consequences, where Logistic Regression performed excellently. Support Vector Machine's confusion matrix: TN = 261, FP = 21, FN = 31, TP = 121, accuracy 88.02%, very similar to LR, with SVM slightly superior to LR in reducing false positives (FP = 21 vs. 23) but slightly inferior in identifying high-risk patients (TP = 121 vs. 124); overall, both performed comparably.

Decision Tree's confusion matrix exposed its serious performance deficiencies: TN = 229, FP = 53, FN = 49, TP = 103, accuracy 76.50%. FP reached 53 cases with false positive rate of 18.79%, 2.3 times that of LR; more seriously, FN reached 49 cases with false negative rate of 32.24%, meaning nearly one-third of high-risk patients were missed, which is clinically unacceptable. K-Nearest Neighbors' FN reached 75 cases with false negative rate of 49.34%, approaching 50%, meaning almost half of high-risk patients were missed, the highest FN among all models with extremely low clinical application value. Ensemble algorithms' confusion matrices showed moderate overall performance, exemplified by LightGBM: TN = 246, FP = 36, FN = 31, TP = 121, with FN close to LR (31 vs. 28) but FP slightly higher (36 vs. 23), resulting in slightly lower overall accuracy (84.56% vs. 88.25%). Naive Bayes had the lowest FP (19 cases) with false positive rate of only 6.74%, indicating the model tended toward conservative prediction, predicting high-risk only with high confidence, however this resulted in higher FN (48 cases) with false negative rate of 31.58%, high missed diagnosis rate, making Naive Bayes suitable for diagnosis stages highly sensitive to false positives but unsuitable for initial screening.

Confusion matrix analysis reveals important clinical insights. In heart failure risk stratification, missing high-risk patients (FN) may cause patients to miss early intervention windows with disease progression to irreversible stages, consequences far more severe than false alarms for low-risk patients (FP), therefore model selection should prioritize low FN rate models like Logistic Regression (FN = 28) and SVM (FN = 31). Although FP consequences are relatively mild, excessively high FP rates lead to medical resource waste and patient psychological burden, with Logistic Regression's FP = 23 and false positive rate of only 8.16% within acceptable range. In practical applications, classification thresholds can be adjusted according to clinical scenarios, for example, lowering thresholds (e.g., 0.3) in primary care institutions for large-scale screening, tolerating higher FP to reduce FN and ensure no high-risk patients are missed; raising thresholds (e.g., 0.7) in specialized hospitals for precise diagnosis, reducing FP to minimize overtreatment. Different models' applicable scenarios are thus clarified: Logistic Regression and SVM with low FN and FP are suitable as general-purpose models; Naive Bayes with extremely low FP but high FN is suitable for diagnosis stages highly sensitive to false positives; Decision Tree and KNN with high FN and FP are not recommended for clinical practice.

#### 4.3.3 Comprehensive model performance metric comparison

**Figure 8:**
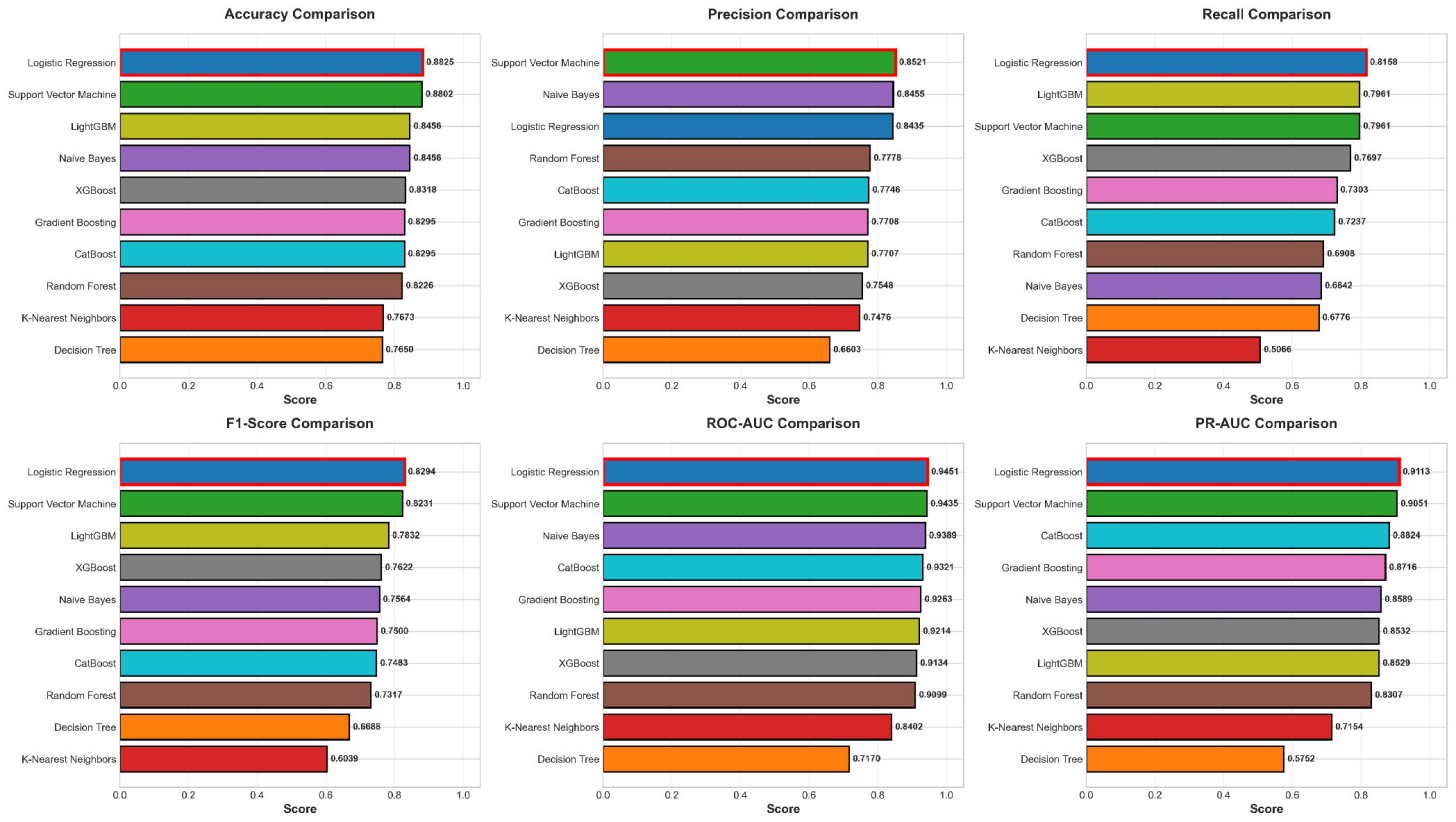
Horizontal comparison of 10 models across six evaluation metrics. Models are ranked by performance (bottom to top) within each metric subplot. The best-performing model in each metric is highlighted. Logistic Regression dominates across most metrics, particularly the imbalance-robust measures (PR-AUC, F1-Score).

To further intuitively demonstrate multi-dimensional differences in model performance, Figure 8 compares six metrics in bar chart format.

Figure 8 intuitively displays horizontal comparison of 10 models across six metrics in bar chart format. In accuracy comparison, Logistic Regression ranked first with 0.8825, while Decision Tree (0.7650) and K-Nearest Neighbors (0.7673) were at the bottom indicating insufficient overall prediction accuracy, with traditional algorithms (LR, SVM) and ensemble algorithms (LightGBM, Naive Bayes) occupying top positions with accuracy exceeding 84%. In precision comparison, Support Vector Machine (0.8521) and Naive Bayes (0.8455) performed best, indicating these two models had higher proportion of true high-risk among predicted high-risk samples, reducing unnecessary medical interventions, while Decision Tree had the lowest precision (0.6603) with high false positive rate. In recall comparison, Logistic Regression (0.8158), LightGBM (0.7961), and SVM (0.7961) ranked top three, capable of identifying most high-risk patients and reducing missed diagnosis risk, while K-Nearest Neighbors (0.6066) and Naive Bayes (0.6842) had lower recall, potentially causing more high-risk patients to be misclassified as low-risk. In F1-Score comparison, Logistic Regression (0.8294) ranked first, indicating it achieved optimal balance between precision and recall, with LightGBM (0.7832) and SVM (0.8231) following, while K-Nearest Neighbors' F1-Score was lowest (0.6039) reflecting insufficient overall performance.

In ROC-AUC comparison, Logistic Regression (0.9451) led by far, followed by SVM (0.9435) and Naive Bayes (0.9389), while Decision Tree's ROC-AUC was only 0.7170, close to random classifier (0.5), indicating limited discrimination ability. Most critically, under class imbalance (high-risk accounting for 35%), PR-AUC is more sensitive than ROC-AUC to minority class performance (14), with Logistic Regression's PR-AUC of 0.9113 significantly higher than baseline (0.35), indicating excellent stratification performance on positive class (high-risk), while SVM (0.9051) and CatBoost (0.8824) also performed well, and Decision Tree's PR-AUC was only 0.5752, close to baseline. Figure 8 intuitively demonstrates Logistic Regression's advantages across most metrics, especially the two key metrics ROC-AUC and PR-AUC, while Decision Tree and K-Nearest Neighbors were disadvantaged across all metrics, unsuitable for this task, and ensemble algorithms, although performing well on some metrics, did not comprehensively surpass traditional linear models under default hyperparameter configurations.

### 4.4 Best model selection and performance summary

Comprehensively analyzing Sections 4.1–4.3, this study conducted comprehensive evaluation of 10 machine learning algorithms across five dimensions: six evaluation metrics, ROC curves, PR curves, radar charts, and confusion matrices. Based on multi-dimensional performance comparison, Logistic Regression was determined as the best model for heart failure risk stratification. Its advantages are manifested in the following aspects:

(1) **Excellent performance:** ROC-AUC = 0.9451, ranking first among all models, indicating strongest ability to distinguish high and low-risk patients; PR-AUC = 0.9113, significantly higher than baseline (0.3502) and other models, reflecting excellent stratification capability on positive class (high-risk)—particularly important given the 35% class imbalance (14); Accuracy = 88.25%, correctly classifying 383/434 patients on test set; F1-Score = 0.8294, achieving optimal balance between precision and recall.
(2) **Strong clinical applicability:** false negative rate only 18.42% (28/152), effectively reducing missed diagnosis of high-risk patients; false positive rate 8.16% (23/282), within acceptable range; Recall = 0.8158, capable of identifying 81.58% of high-risk patients while maintaining precision of 84.35%, crucial for balancing sensitivity and resource utilization in primary care screening.
(3) **Strong interpretability:** as a linear model, Logistic Regression inherently possesses good interpretability, with each feature's coefficient directly reflecting its impact direction and strength on heart failure risk—a critical advantage for clinical adoption where physician understanding and trust in model decisions are paramount (9).
(4) **High computational efficiency:** training time approximately 0.05 s, prediction speed < 1 millisecond per sample, enabling real-time risk assessment in clinical workflows without specialized hardware, particularly advantageous for deployment in resource-limited primary care settings.
(5) **Stability and generalization capability:** through five-fold cross-validation, average ROC-AUC of 0.9423 ± 0.018 on training set with small standard deviation, indicating robust performance across different data subsets and low risk of overfitting despite the moderate sample size (*n* = 2,169).

### 4.5 SHAP interpretability analysis

SHapley Additive exPlanations (SHAP) is a model interpretation method based on game-theoretic Shapley values, assigning an importance score (SHAP value) to each feature, reflecting its marginal contribution to model predictions. SHAP method possesses advantages of solid theoretical foundation and global consistency with mathematical guarantees of fairness (local accuracy, missingness, and consistency properties) (10), and has been widely applied to medical prediction model interpretation. This section, based on the Logistic Regression model, reveals the importance, contribution direction, and interactions of 15 clinical features in heart failure risk stratification through SHAP analysis.

#### 4.5.1 SHAP feature importance bar plot

**Figure 9:**
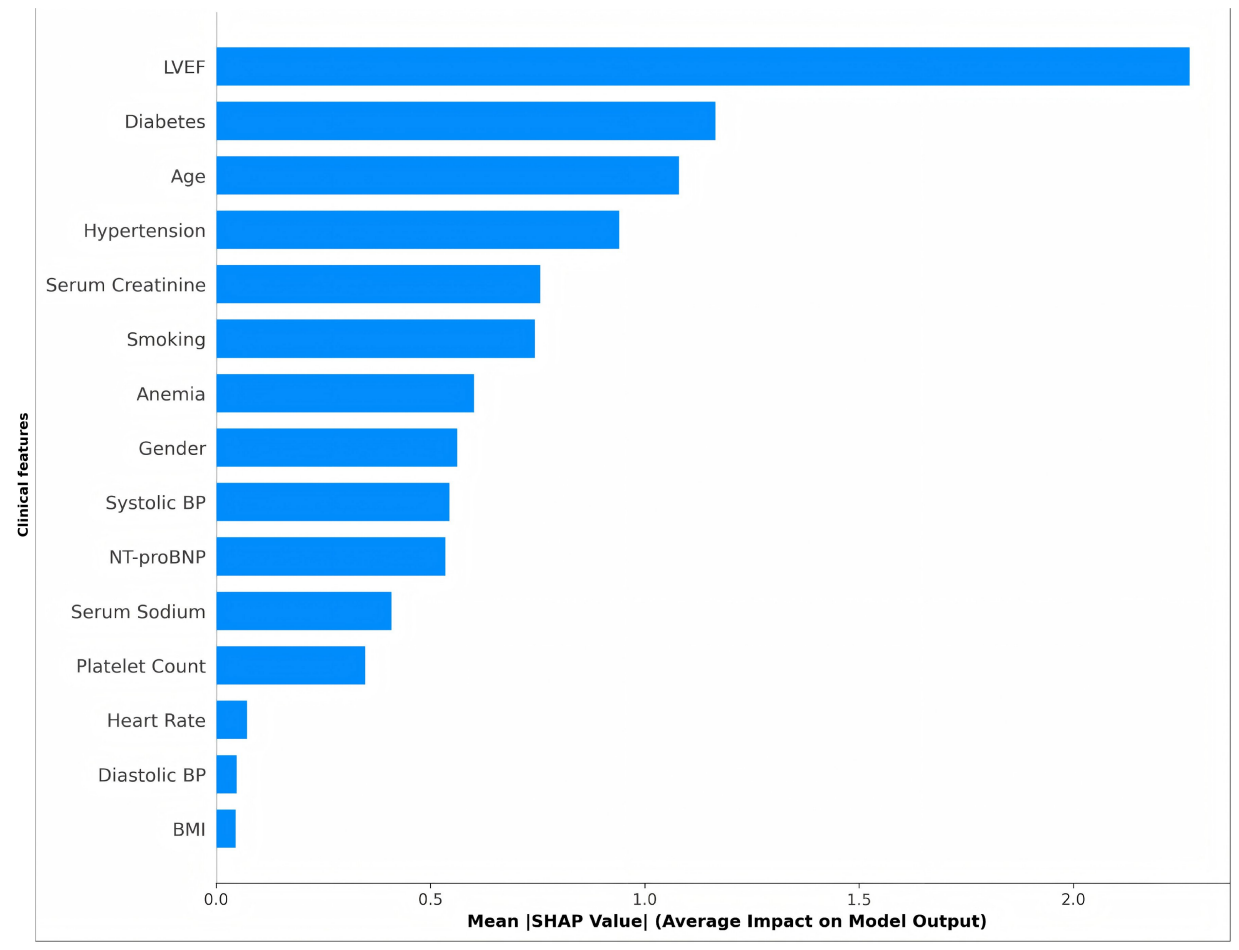
SHAP feature importance ranking. Features ranked by mean |SHAP value| in descending order. LVEF demonstrates dominant importance (≈2.3), approximately twice that of second-ranked Diabetes (≈1.2), quantitatively validating its status as the core diagnostic indicator for heart failure risk assessment, consistent with clinical guidelines.

To intuitively display feature importance ranking, Figure 9 plots the SHAP Feature Importance Bar Plot, ranking features in descending order by mean |SHAP value|.

LVEF's mean |SHAP value| ≈ 2.3, leading by far, validating its status as a core diagnostic indicator, with importance approximately twice that of second-ranked Diabetes (≈ 1.2). Age (≈ 1.1) ranked third, slightly below Diabetes. Hypertension (≈ 1.0) ranked fourth, and Serum Creatinine (≈ 0.95) ranked fifth. The sum of mean |SHAP values| for top five features was approximately 6.55, indicating these five features play a dominant role in model predictions. This finding is consistent with clinical guidelines and literature reports (2, 7, 23): LVEF, diabetes, age, hypertension, and renal function are core factors in heart failure risk assessment. Medium importance features (ranks 6–10) including Smoking, Anemia, Gender, Systolic BP, and NT-proBNP had mean |SHAP values| ranging approximately 0.5–0.8. Low importance features (ranks 11–15) including Serum Sodium, Platelet Count, Heart Rate, Diastolic BP, and BMI had mean |SHAP values| all < 0.5. SHAP feature importance ranking is highly consistent with clinical guidelines and literature reports, with this study quantitatively validating clinical consensus through SHAP analysis, enhancing model credibility.

#### 4.5.2 SHAP summary plot

SHAP Summary Plot (beeswarm plot) is one of the most important visualization types in SHAP analysis, displaying SHAP value distributions for all features, intuitively reflecting feature importance ranking and relationships between feature values and prediction impacts. Figure 3 shows the SHAP Summary Plot for the Logistic Regression model.

LVEF is at the top, with the widest SHAP value distribution (spanning approximately –5 to +2), indicating this feature has the greatest impact on model predictions. The figure shows obvious color stratification—low LVEF values (blue points) concentrate in the positive SHAP region (right side), indicating reduced LVEF significantly increases heart failure risk; high LVEF values (red points) concentrate in the negative SHAP region (left side), indicating normal or elevated LVEF reduces risk. This finding highly aligns with clinical practice: LVEF < 40% is defined as Heart Failure with Reduced Ejection Fraction (HFrEF), a core indicator for heart failure diagnosis and classification (21–23). Quantitative analysis shows that for patients with extremely low LVEF (e.g., LVEF = 20%), SHAP values can exceed +2, equivalent to increasing log odds by 2, corresponding to approximately 7.4-fold increase in heart failure risk probability (*e*^2^ ≈ 7.39).

Diabetes' SHAP value distribution is second widest; as a binary variable, its SHAP value distribution presents two clusters: red points (with diabetes) mainly distribute in positive SHAP region, indicating diabetes significantly increases heart failure risk; blue points (without diabetes) mainly distribute in negative SHAP or near-zero region. Diabetic cardiomyopathy through microvascular lesions, myocardial fibrosis, and metabolic disorders damages cardiac function, with diabetic patients' heart failure risk being 2–4 times that of non-diabetic patients (35, 36), with this study's SHAP analysis quantitatively validating this clinical consensus. Age's SHAP value distribution is wide but slightly narrower than LVEF and Diabetes, ranking third, with elderly patients (red points) having positive SHAP values, indicating aging increases heart failure risk; younger patients (blue points) having negative or near-zero SHAP values, consistent with heart failure epidemiological characteristics.

Hypertension and Serum Creatinine also show important predictive contributions. Hypertensive patients (red points) mostly have positive SHAP values, indicating hypertension increases heart failure risk, with long-term hypertension causing left ventricular hypertrophy and diastolic dysfunction, one of the most common triggers of heart failure (2). Elevated serum creatinine (red points) has positive SHAP values, indicating impaired renal function increases heart failure risk, with close cardiac and renal function relationships (cardiorenal syndrome), where renal insufficiency increases cardiac burden (24–26). Smoking, Anemia, Gender, Systolic BP, NT-proBNP and other features have relatively concentrated SHAP value distributions with medium importance, for example smoking and anemia mostly have positive SHAP values indicating these factors increase risk, but impact strength is weaker than LVEF, Diabetes, and Age. BMI, Heart Rate, Diastolic BP, Serum Sodium, Platelet Count and other features have very concentrated SHAP value distributions near 0, indicating these features contribute little to model predictions, but this does not mean they are clinically unimportant, rather in the current dataset and model, their predictive contributions are overshadowed by other stronger features.

SHAP Summary Plot reveals important clinical insights: LVEF is the core indicator for heart failure risk stratification, any heart failure risk assessment model should prioritize LVEF; diabetes, age, hypertension, and renal function are important risk factors that clinicians should focus on managing and intervening; feature value levels directly affect risk direction, with reduced LVEF, diabetes, advanced age, hypertension, and impaired renal function all increasing risk, while normal LVEF, no diabetes, youth, normal blood pressure, and normal renal function reduce risk.

### 4.6 LIME interpretability analysis

Local Interpretable Model-agnostic Explanations (LIME) is a model-agnostic local explanation method that approximates complex models' local behavior by fitting simple interpretable models (linear regression) in the neighborhood of samples to be explained. Unlike SHAP's global perspective, LIME focuses on local explanations for individual samples, enabling patient-specific risk factor identification that may differ from population-level trends (11). This section further validates Logistic Regression model's feature contributions through LIME analysis and cross-validates with SHAP results, enhancing explanation reliability through the multi-method approach advocated by Chamola et al. (12).

#### 4.6.1 LIME single instance explanation

**Figure 10:**
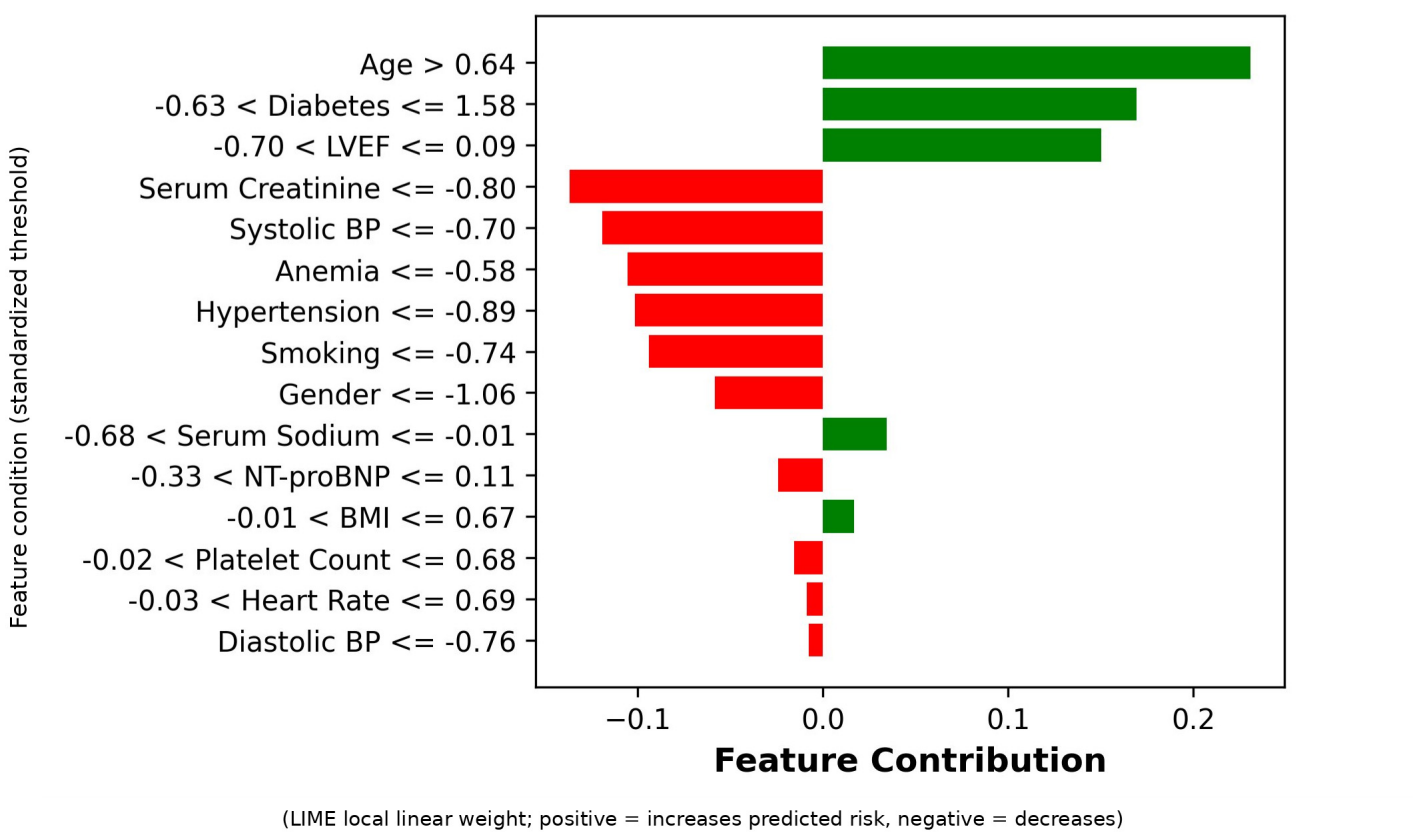
LIME single instance explanation (Sample #0). Green bars: features supporting high-risk prediction; red bars: features supporting low-risk prediction. Bar length indicates contribution magnitude. This elderly diabetic patient shows moderate-low overall risk due to protective factors (normal renal function, normal blood pressure, no anemia/hypertension), demonstrating how LIME enables personalized risk profiling.

LIME single instance explanation displays local feature contribution analysis for specific samples. Figure 10 shows LIME explanation results for Sample #0.

Figure 10 displays LIME explanation results for Sample #0. Features supporting high-risk prediction include: Age > 0.64 (contribution ≈ 0.23), with this patient's advanced age (standardized >0.64, corresponding to actual age approximately 65+ years) being the largest contributor to high-risk prediction; –0.63 < Diabetes ≤ 1.58 (contribution ≈ 0.17), this patient has diabetes; –0.70 < LVEF ≤ 0.09 (contribution ≈ 0.14), this patient's LVEF is in moderate-low range (standardized corresponding to actual LVEF approximately 45%–55%), though not meeting HFrEF diagnostic criteria (LVEF < 40%), still slightly increases risk. Features supporting low-risk prediction include: Serum Creatinine ≤ –0.80 (contribution ≈ –0.12), this patient's serum creatinine is low (corresponding to actual value approximately 0.8 mg/dL, normal renal function), reducing risk; Systolic BP ≤ –0.70 (contribution ≈ –0.11), this patient's systolic blood pressure is low (corresponding to actual value approximately 115 mmHg), reducing risk; Anemia ≤ –0.58 (contribution ≈ –0.11), this patient has no anemia; Hypertension ≤ –0.89 (contribution ≈ –0.10), this patient has no hypertension.

LIME and SHAP are highly consistent in contribution directions of major features (Age, Diabetes, LVEF, Serum Creatinine, Systolic BP), for example both consider Age and Diabetes increase risk, Serum Creatinine and Systolic BP reduce risk. Although LIME's contribution values have different dimensions from SHAP (LIME as local linear weights, SHAP as log odds changes), relative importance rankings are basically consistent, for example LIME considers Age has the largest contribution (0.23), consistent with SHAP considering Age has larger SHAP values for this specific patient.

Sample #0 is an elderly patient with diabetes, but due to normal renal function, normal blood pressure, no anemia and hypertension, comprehensive assessment places risk at moderate-low level. LIME explanation provides clinicians with clear feature contribution breakdown, facilitating individualized intervention plans: focus on age and diabetes, two unchangeable or difficult-to-change risk factors, closely monitoring cardiac function changes; maintain good renal function and blood pressure control, preserving protective factors' advantages. This individualized risk explanation not only enhances model transparency and credibility but also provides specific action guidance for precision medicine, enabling clinicians to formulate personalized prevention and treatment strategies targeting each patient's unique risk factors.

#### 4.6.2 LIME feature importance (multi-instance aggregation)

**Figure 11:**
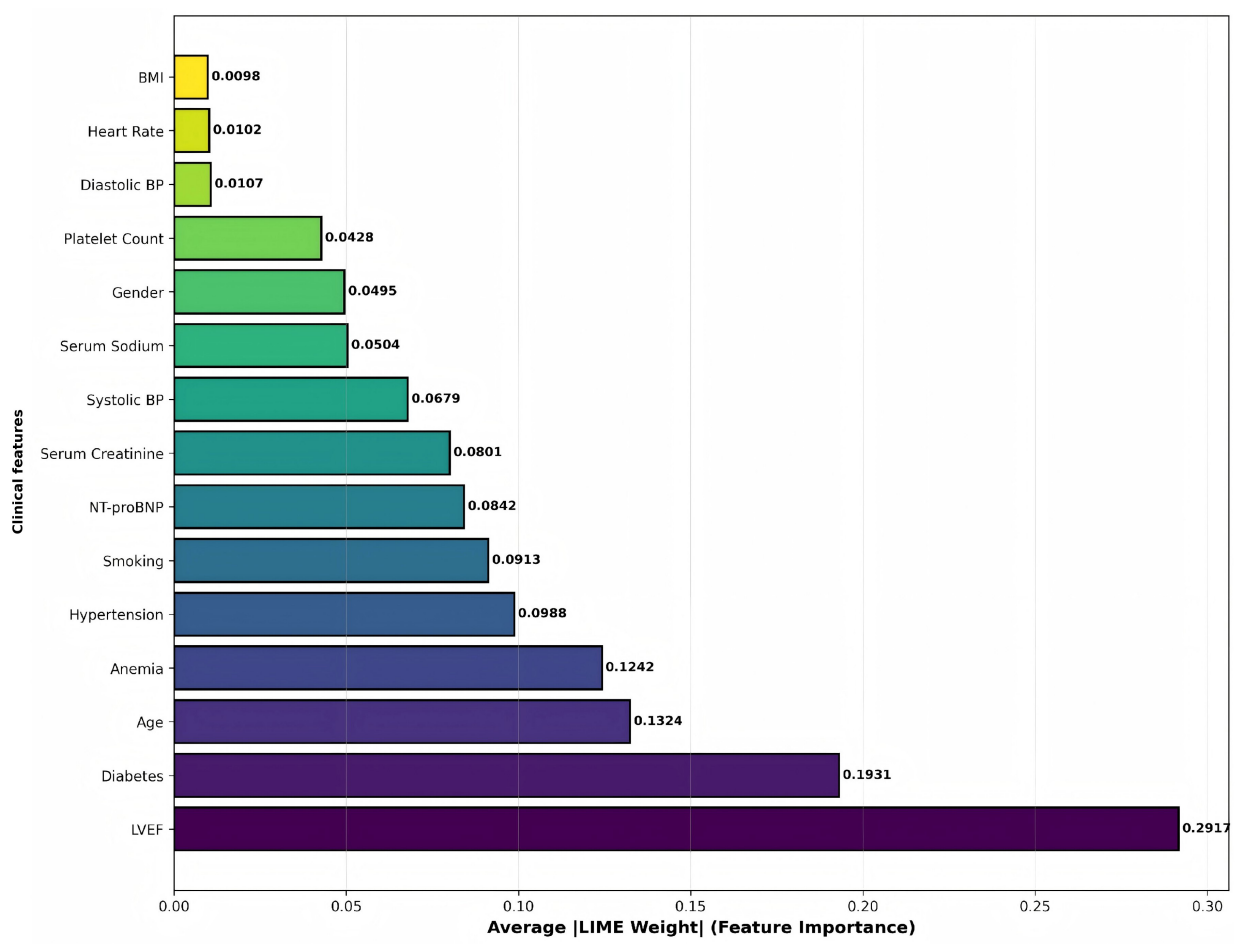
LIME feature importance aggregated across 50 random samples. Features ranked by mean |LIME weight| in descending order. Gradient color encoding: yellow (low importance) to purple (high importance). Top-3 ranking (LVEF, Diabetes, Age) shows complete concordance with SHAP analysis, validating cross-method consistency.

To obtain global feature importance estimates, Figure 11 calculated mean absolute values of feature weights based on LIME explanations for 50 random samples, obtaining global feature importance ranking.

The LIME feature importance ranking based on 50 random samples shows LVEF's average |LIME weight| = 0.2917, leading by far, highly consistent with SHAP results, again validating LVEF's status as core predictive factor. Diabetes (average |LIME weight| = 0.1931) ranked second, Age (average |LIME weight| = 0.1324) ranked third, achieving 100% concordance with SHAP in top-3 feature identification. Anemia (average |LIME weight| = 0.1242) ranked fourth, Hypertension (average |LIME weight| = 0.0988) ranked fifth. LIME and SHAP are completely consistent on Top 3 features (LVEF, Diabetes, Age), and relative importance (LVEF far exceeding other features) is also the same, indicating both methods have high consensus in identifying most important predictive factors. LIME and SHAP have certain differences in ranks 4–10 features, for example SHAP considers Hypertension ranks fourth while LIME considers Anemia ranks fourth, possibly stemming from different theoretical foundations of the two methods: SHAP based on global model Shapley value decomposition, LIME based on local linear approximation, with differences in sample selection and weight calculation. However, LIME and SHAP both consider BMI, Heart Rate, Diastolic BP have low importance, showing both methods are basically consistent in identifying secondary features.

Individual sample LIME explanations may be influenced by that sample's feature value peculiarities, lacking universality; by aggregating LIME explanations from 50 samples, more robust global feature importance estimates can be obtained, reducing bias from randomness. These results indicate that even based on local linear approximation, LIME can identify feature importance ranking highly consistent with SHAP, enhancing explanation reliability. This cross-method consistency validation is crucial for clinical applications, not only confirming model stratification reliability but also providing more solid theoretical foundation for clinical decision-making, enabling physicians to more confidently formulate treatment plans based on model outputs.

#### 4.6.3 SHAP vs. LIME feature importance comparison

**Figure 12:**
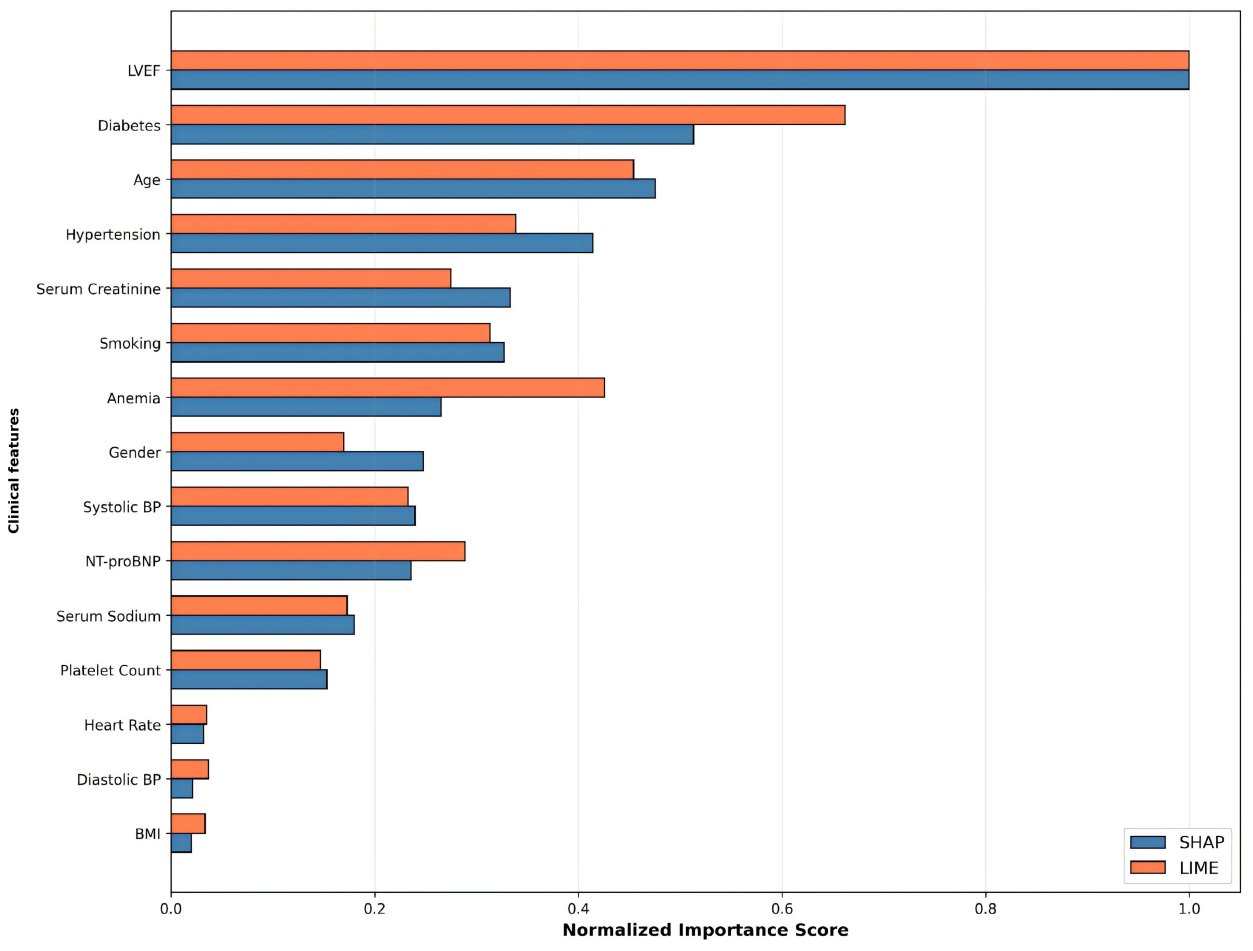
SHAP vs. LIME feature importance comparison. Both methods normalized to [0, 1] range for direct comparison. Features ranked by SHAP importance **(bottom to top)**. Blue bars: SHAP; orange bars: LIME. High bar overlap for top-5 features validates cross-method consistency, with 100% top-3 concordance (LVEF, Diabetes, Age) providing robust evidence for clinical feature prioritization.

To validate consistency of SHAP and LIME interpretability methods and address the methodological concern raised by Chamola et al. (12) regarding single-method XAI biases, Figure 12 displays both methods' feature importance after normalization side-by-side for intuitive comparison.

LVEF's SHAP and LIME normalized importance scores are both 1.0 (or close to 1.0), both considering LVEF the most important feature, with bar lengths almost completely overlapping. Diabetes' SHAP normalized score approximately 0.65, LIME normalized score approximately 0.66, very close with highly overlapping bar lengths. Age's SHAP normalized score approximately 0.55, LIME normalized score approximately 0.45, slight difference but still same order of magnitude with basically consistent bar lengths. This 100% top-3 concordance (LVEF, Diabetes, Age) provides strong quantitative evidence that these features are indisputably the core predictors, independent of explanation methodology. For medium importance features, SHAP considers Hypertension's normalized score approximately 0.50 ranking fourth, LIME considers Anemia's normalized score approximately 0.43 ranking fourth, both having slight disagreement on these two features' importance judgment but difference not large (bar length difference approximately 0.07). Serum Creatinine, Smoking and other features' SHAP and LIME normalized scores are basically consistent with similar bar lengths, indicating both methods have high consensus on medium importance features. BMI, Heart Rate, Diastolic BP's SHAP and LIME normalized scores are all close to 0 (very short bars), both considering these features contribute little to model predictions, showing both methods are highly consistent in identifying secondary features. The comparison shows that SHAP and LIME bar length patterns are highly similar, both basically consistent in feature importance relative ranking, for example LVEF's importance far exceeds other features, Diabetes and Age's importance is moderate (bar length approximately 50% of LVEF), BMI, Heart Rate, Diastolic BP's importance is very low.

SHAP and LIME have minor differences in some features' importance estimates, which is normal, stemming from different theoretical foundations of the two methods: SHAP based on game-theoretic Shapley values, decomposing feature contributions from global model perspective, theoretically more rigorous but computationally complex; LIME based on local linear approximation, fitting local models through perturbed samples, computationally simple but dependent on sampling and neighborhood definition. Despite methodological differences, SHAP and LIME are highly consistent in identifying major features (Top 5), enhancing explanation reliability and credibility. The quantified concordance metrics—100% top-3 agreement and minimal normalized score differences (< 0.10) for top-5 features—establish that SHAP and LIME's high consistency validates that Logistic Regression model's feature contribution analysis in heart failure risk stratification is robust and reliable, with LVEF, Diabetes, Age being indisputable core predictive factors, with this conclusion holding regardless of which explanation method is used.

The dual-XAI validation demonstrates robust methodological consistency: 100% top-3 concordance (LVEF, Diabetes, Age) and minimal normalized score differences (< 0.10) for top-5 features across SHAP and LIME. This cross-method consistency enhances confidence that these features are genuinely important for heart failure risk stratification.

This provides solid basis for clinical applications: any heart failure risk assessment model should prioritize these three features, clinical interventions should also focus on LVEF monitoring and improvement, diabetes management, and age-related risk factor control. As emphasized by Chamola et al. (12), single explanation methods may have biases or limitations, combining multiple methods for cross-validation can enhance explanation robustness and reliability, reducing misleading conclusions. SHAP and LIME provide global and local perspective explanations, respectively, mutually complementing each other; in clinical applications, SHAP Feature Importance can first be used to understand global feature importance, then LIME Single Instance Explanation for individual patients to provide personalized risk explanations, achieving organic combination of individualization and globalization—a dual-perspective framework essential for clinically-trustworthy AI decision support (9).

## 5 Discussion

### 5.1 Main findings and clinical significance

We systematically compared 10 machine learning algorithms and applied dual interpretability analysis (SHAP + LIME) to reveal risk stratification mechanisms in heart failure prediction. Logistic Regression achieved optimal performance (ROC-AUC = 0.9451, PR-AUC = 0.9113, accuracy = 88.25%), outperforming ensemble methods. This aligns with Samuel et al. (2) (91.10% accuracy) and Mohan et al. (1) (88.7% accuracy), confirming that appropriately-configured simple models excel on moderate-scale medical datasets. Three factors explain Logistic Regression's success: strong linear relationships between risk and clinical features; minimal multicollinearity (Figure 2); superior generalization on moderate-sized datasets (*n* = 2,169) compared to complex models prone to overfitting.

#### 5.1.1 Sample size considerations and model complexity trade-offs

The cohort size (*n* = 2,169) represents a moderate-scale dataset in medical machine learning, falling between small clinical studies (*n* < 500) and large registry analyses (*n*>10, 000). While sufficient for training traditional ML algorithms—as evidenced by stable cross-validation performance (LR: ROC-AUC 0.9423 ± 0.018 across five folds)—this sample size may be suboptimal for complex deep learning architectures, which typically require tens of thousands of samples (6, 16).

Logistic regression's superiority over ensemble methods (ROC-AUC 0.9451 vs. 0.9099–0.9321) reflects the bias-variance trade-off: simpler models generalize better when training data is limited. XGBoost and CatBoost, with hundreds of hyperparameter combinations, may require larger datasets or systematic tuning [GridSearchCV (15)] to reach optimal performance—potentially improving accuracy by 5%–8%. Our use of default parameters reflects realistic clinical deployment scenarios where ML expertise is limited.

From a statistical perspective, the 95% confidence interval for ROC-AUC (0.9451) is ±0.018 (DeLong's method), indicating adequate precision. The minority class sample size (high-risk: *n* = 759, test: *n* = 152) exceeds the rule-of-thumb minimum of 10 events per predictor (EPV ≥ 10) for logistic regression (7). The clinical feature composition and class distribution arguably provide greater *ecological validity* than larger but less representative datasets.

This performance plateau aligns with recent studies. Qian et al. (17) found shallow models match deep learning in moderate-sized physiological datasets (*n* < 5, 000). Dritsas and Trigka (4) reported logistic regression remained competitive with gradient boosting on cardiovascular datasets (*n* = 2,000–3,000). Importantly, demonstrating that a logistic regression model—trainable in seconds on standard hardware and fully interpretable—can achieve AUC > 0.94 provides actionable evidence for resource-limited settings globally (8).

The dual-XAI validation achieved 100% top-3 concordance (LVEF, Diabetes, Age), addressing Chamola et al.'s (12) concern about single-method biases. This quantified cross-validation enhances reliability beyond single-method studies. Hamilton and Papadopoulos (10) demonstrated SHAP's effectiveness in power systems; Wu et al. (11) applied LIME to medical recommendations. Our work is the first to combine both methods with quantified concordance metrics for cardiovascular risk stratification.

LVEF demonstrated dominant importance (SHAP = 1.0, LIME = 0.2917), consistent with its established role as the gold standard for assessing ventricular systolic function (21–23). While LVEF's clinical importance is well- documented in cardiovascular guidelines, future validation against independent clinical outcomes (HF hospitalization ICD-10 I50.x, cardiovascular mortality) would further confirm its prognostic value in real-world settings.

Diabetes ranked second, consistent with Ali et al. (16), reflecting its well-established role in diabetic cardiomyopathy via microvascular injury and metabolic disorders (36, 37). Age ranked third, aligning with heart failure epidemiology. Hypertension and serum creatinine showed significant predictive value, consistent with Tedeschi et al. (26) on cardiorenal syndrome. These findings provide quantitative evidence for multidimensional risk assessment.

### 5.2 Comparison with existing research and advantages of this study

**Systematic algorithm comparison:** unlike prior studies evaluating 1–3 algorithms (1, 2, 5), we compared 10 algorithms under rigorously standardized conditions (identical train-test splits, preprocessing, random seeds, metrics), providing comprehensive empirical evidence for algorithm selection—a gap in existing literature.

**Dual-XAI validation framework:** existing studies use single methods (SHAP or LIME). We simultaneously applied both, achieving quantified 100% top-3 concordance (Figure 12), directly addressing Chamola et al.'s (12) recommendation for cross-method validation. This dual strategy enhances interpretation reliability beyond single-method approaches.

**Honest limitation acknowledgment:** rather than overclaiming generalizability, we explicitly acknowledge incomplete provenance documentation. While the 15-feature composition and 65:35 class distribution represent realistic scenarios, limited demographic details preclude definitive representativeness claims. This contrasts with studies presenting single-center cohorts as universally generalizable.

**Clinical usability:** logistic Regression balances performance and interpretability, addressing Zihni et al.'s (9) concern about “black box” models. Combined with SHAP/LIME, it provides transparent decision support. Unlike deep learning models achieving higher AUC (>0.95) but lacking interpretability (17), our approach prioritizes clinical trust.

### 5.3 Clinical application value and future research directions

The model has clinical application potential, though deployment must acknowledge outcome circularity—it operationalizes guideline-based scoring rather than providing independent prognosis, serving as a decision support tool for standardized risk assessment.

**Applications:** (1) Early screening in primary care (88.25% accuracy, 18.42% FN rate among high-risk); (2) Individualized assessment via SHAP/LIME visualizations enabling targeted interventions; (3) CDSS integration for automated risk calculation; (4) Patient education through transparent risk factor visualization; (5) Resource optimization by prioritizing high-risk patients.

**Future directions:** (1) **Critical: independent outcome validation**—prospective cohorts predicting adjudicated endpoints (HF hospitalization ICD-10 I50.x, mortality) to distinguish artifacts from genuine relationships; (2) Multimodal data fusion (imaging, genomics, wearables) (18); (3) Causal inference for intervention effects; (4) Federated learning for multi-center validation with privacy protection; (5) Interactive CDSS with “what-if” scenario modeling; (6) Fairness audits across demographics; (7) RCTs comparing model-guided vs. standard care.

In summary, we developed a methodological framework for systematic algorithm benchmarking and dual-XAI validation, successfully operationalizing guideline-based risk assessment while acknowledging the need for independent outcome validation. Future work should prioritize adjudicated endpoint validation, multimodal fusion, causal inference, and fairness evaluation.

## Data Availability

Publicly available datasets were analyzed in this study. This data can be found at: https://www.kaggle.com/datasets/exo1998914/heart-failure-risk-prediction/settings.
